# The Role of Mislocalized Phototransduction in Photoreceptor Cell Death of Retinitis Pigmentosa

**DOI:** 10.1371/journal.pone.0032472

**Published:** 2012-04-02

**Authors:** Takeshi Nakao, Motokazu Tsujikawa, Shoji Notomi, Yasuhiro Ikeda, Kohji Nishida

**Affiliations:** 1 Department of Ophthalmology, Osaka University Graduate School of Medicine, Suita, Osaka, Japan; 2 Department of Ophthalmology, Graduate School of Medical Sciences, Kyushu University, Fukuoka, Japan; University of Oldenburg, Germany

## Abstract

Most of inherited retinal diseases such as retinitis pigmentosa (RP) cause photoreceptor cell death resulting in blindness. RP is a large family of diseases in which the photoreceptor cell death can be caused by a number of pathways. Among them, light exposure has been reported to induce photoreceptor cell death. However, the detailed mechanism by which photoreceptor cell death is caused by light exposure is unclear. In this study, we have shown that even a mild light exposure can induce ectopic phototransduction and result in the acceleration of rod photoreceptor cell death in some vertebrate models. In *ovl*, a zebrafish model of outer segment deficiency, photoreceptor cell death is associated with light exposure. The *ovl* larvae show ectopic accumulation of rhodopsin and knockdown of ectopic rhodopsin and transducin rescue rod photoreceptor cell death. However, knockdown of phosphodiesterase, the enzyme that mediates the next step of phototransduction, does not. So, ectopic phototransduction activated by light exposure, which leads to rod photoreceptor cell death, is through the action of transducin. Furthermore, we have demonstrated that forced activation of adenylyl cyclase in the inner segment leads to rod photoreceptor cell death. For further confirmation, we have also generated a transgenic fish which possesses a human rhodopsin mutation, Q344X. This fish and *rd10* model mice show photoreceptor cell death caused by adenylyl cyclase. In short, our study indicates that in some RP, adenylyl cyclase is involved in photoreceptor cell death pathway; its inhibition is potentially a logical approach for a novel RP therapy.

## Introduction

Retinitis pigmentosa (RP, MIM 26800) is a common group of inherited retinal diseases that lead to blindness. RP affects 19–27 out of 100,000 people all over the world [1], [2]. Usually, patients first suffer from peripheral visual field loss because of peripheral rod photoreceptor cell death. Photoreceptor cell death gradually but steadily progresses until patients lose central visual function, which degrades quality of life. Such slow progressive photoreceptor cell death is a prominent feature of RP.

RP shows typical locus heterogeneity. More than 45 responsible genes have been identified, however, the detailed mechanism of progressive photoreceptor cell death is still entirely unknown (RetNet, http://www.sph.uth.tmc.edu/retnet/), [3]–[7]. A set of cilium genes are thought to be responsible for Bardet–Biedl syndrome (BBS, MIM 209900), a systemic syndrome that includes RP and kidney cysts [8]–[13]. Recently, we identified zebrafish photoreceptor mutant *oval (ovl)*, which encodes one such cilium gene, IFT88. It's mutation causes mislocalized visual pigment and cilia dysfunction, leading to the loss of outer segments [14].

**Figure 1:**
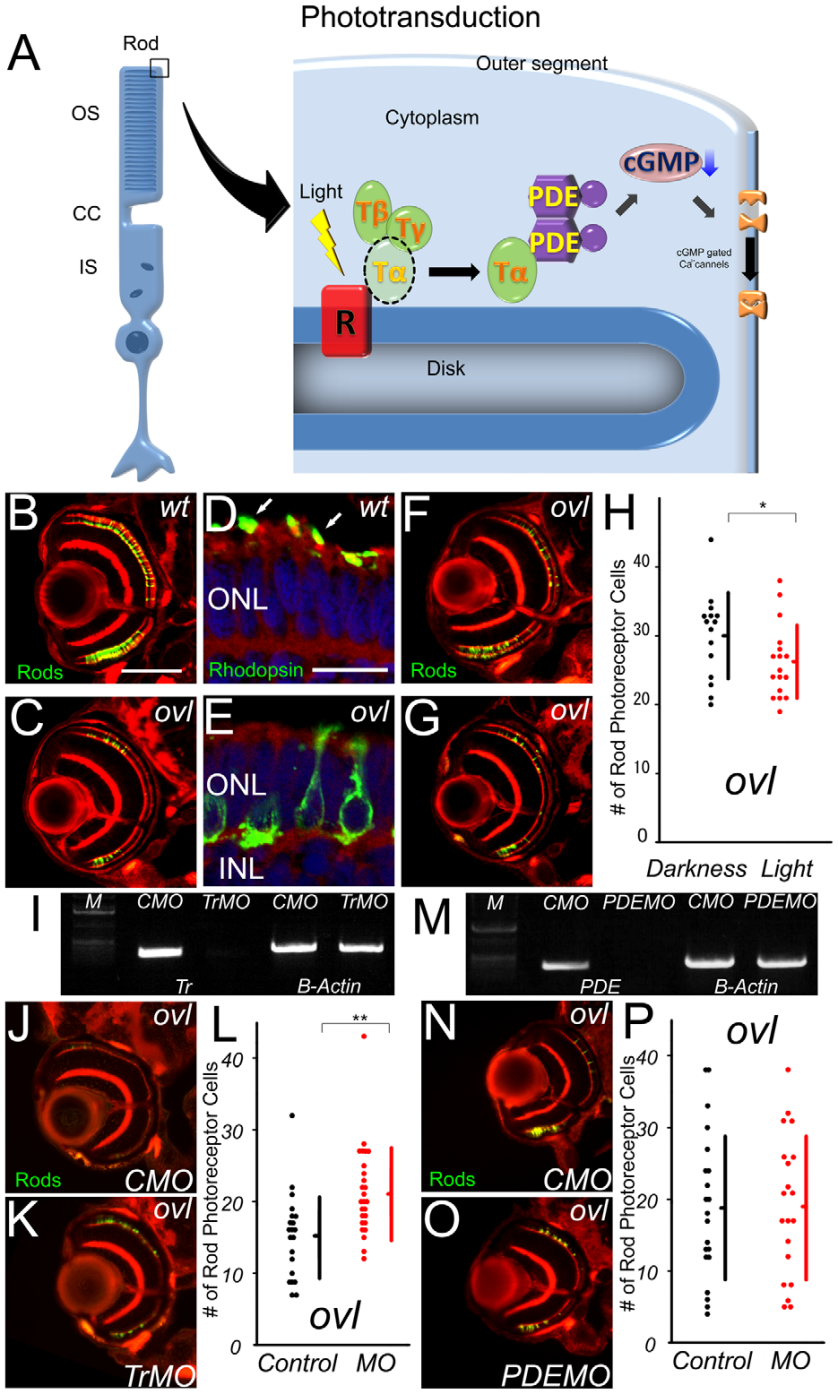
The pathway of photoreceptor cell death does not include PDE6β. (A) Schematic diagram of Phototransduction cascade. OS: outer segment, CC: connecting cilium, IS: inner segment, R: rhodopsin, T: transducin, PDE: phosphodiesterase (B and C) Eye ections of eyes from wt (B) and *ovl* (C) fish at 108 hpf. Rod photoreceptors are visualized with EGFP (Green). (Bar = 100 µm.) In *ovl*, the number of rod photoreceptors was decreased. (D and E) Transverse cryosections through wt (D) and *ovl* mutant (E) retinae at 4 dpf. F-actin is visualized with phalloidin (red), rod opsin with antibodies (green) and nuclei with Hoechst33342 (blue). Rhodopsin is mis-localized in *ovl*. Arrows indicate outer segments. (Bar = 10 µm.) ONL: outer nuclear layer, INL: inner nuclear layer (F and G)*Ovl* animals were reared in constant darkness (F) or in constant light (G) at 108 hpf. Light exposure reduces the survival of rod photoreceptor cells. (H) Graph of the number of rod photoreceptors in *ovl* fish at 108 hpf. (Bars mean SD, * means p<0.05.) (I) Trandsucin α expression analysis by RT-PCR of control morpholino- (lane1) and anti-Transducin α morpholino-treated (lane2). The expression of transducin is effectively suppressed by the morpholino at 108 hpf. CMO: control morpholino, TrMO: anti-Transducin α morpholino, (J and K) Sections of eyes treated by anti-transducin α morpholinos (K) and control MO (J) in *ovl* at 108 hpf. Anti-transducin a morpholinos rescued the rod photoreceptor cell death. (L) Rod photoreceptor numbers in anti-transducin α and control morpholino-treated *ovl* mutants at 108 hpf. (Bars mean SD, ** means p<0.01.) (M) PDE6β expression analysis by RT-PCR of control morpholino- (lane1) and anti-PDE6β (lane2) morpholino-treated mutants. The expression of PDE6β is effectively suppressed by the morpholino at 108 hpf. (N and O) Sections of eyes treated by anti-phosphodiesterase 6β morpholinos (O) and control MO (N) in *ovl*. (Bar = 100 µm.) There are no significant difference. (P) Rod photoreceptor survival in anti-phosphodiesterase 6β morpholino- (red dots) and control (black dots) morpholino-treated *ovl* mutants. Anti-PDE 6β morpholino has no significant effect on the number of rod photoreceptors. (Bars mean SD.)

This mislocalization may play an important role in human disease because it is observed in human photoreceptor cell death caused by RP, retinal detachment [15], [16], and age-related macular degeneration [17]. We have also shown that the mislocalization causes ectopic phototransduction, which accelerates photoreceptor cell death in *ovl* [14]. However the mechanism of photoreceptor cell death caused by the mislocalization is entirely unknown. Phototransduction is the process of a cell absorbing light and creating a response. In this pathway the photoreceptor-specific G protein, transducin, mediates between the light receptor, rhodopsin, and the effecter enzyme, phosphodiesterase (Figure 1A). In this article, we demonstrate a detailed mechanism of photoreceptor cell death coupled with mislocalized phototransduction via transducin.

## Results

### Light exposure accelerates rod photoreceptor cell death

To investigate the relationship between photoreceptor cell death and phototransduction, we assessed whether the photoreceptor cell death in *ovl* is accelerated by phototransduction. In *ovl*, rod photoreceptor cells degenerate progressively (Figure S1). At 108 hours post fertilization (hpf), the degeneration of rod photoreceptors in *ovl* is obvious (Figure 1B, C). As human retinitis pigmentosa, the degeneration is predominantly shown in rod photoreceptor and, cone photoreceptors do not degenerate severely in early stage. (Figure S2). And the *ovl* retina does not develop an outer segment, resulting in mislocalized photopigments, which mimics many human photoreceptor diseases (Figure 1D, E) [9], [15], [16]. We first confirmed whether rod photoreceptor cell death in *ovl* is light dependent as we previously reported [14]. The number of rod photoreceptor cells showed a significant difference (p<0.0378) between the continuous light exposure group (n = 17, average = 26.2) and control group (n = 15, average = 30.1) of *ovl* mutants at 108 hpf (Figure 1F–H). The exposure did not influence the number of wild-type rod photoreceptors (light exposure group; n = 13, average = 65.2 and control group; n = 11, average = 66.1, p = 0.269). In addition we have shown that morpholino knock down of mislocalized rhodopsin rescues *ovl* photoreceptor cell death [14]. These results strongly suggest that early mislocalized phototransduction leads to photoreceptor cell death.

### Transducin α in phototransduction cascade is involved in rod photoreceptor cell death

For further investigation of the role of the phototransduction cascade in rod photoreceptor cell death, we shut down phototransduction using a morpholino knock down of transducin in normal light cycle condition. We used anti-transducin α morpholinos which effectively down-regulated transducin α transcription at 108 hpf (Figure 1I). Suppression of transducin α significantly increased the number of surviving rod photoreceptor cells in the morpholino-treated *ovl* retina 108 hpf (n = 26, average = 21.3), compared to the control (n = 21, average = 15.1, p<6.68E-4). Over 35% of the rods survived in transducin α morpholino-treated animals (Figure 1J–L). On the other hand, in wild-type animals the number of surviving rod photoreceptor cells is equivalent (p = 0.265) between morpholino treated (n = 23, average = 32.7) and control (n = 25, average = 33.4) animals (data not shown). TUNEL analysis revealed that apoptosis in outer nuclear layer in *ovl* is decreased by transducin α morpholino treatment (Figure S3). These results indicate that transducin α, in addition to rhodopsin, is involved in the rod photoreceptor cell death pathway in *ovl*.

### Suppression of phosphodiesterase 6β does not affect the rod photoreceptor cell death

We then tested the contribution of the rod cGMP-phosphodiesterase β subunit (PDE6β), the step following transducin during phototransduction in rod photoreceptor cell death. Injection of PDE6β morpholino effectively suppressed PED6β expression (Figure 1M), however, it did not affect the number of surviving rod photoreceptor cells in the morpholino-treated *ovl* retina (n = 20, average = 19.1, Figure 1N) at 108 hpf, compared to control morpholino-treated mutants (n = 21, average = 18.9; Figure 1O, p = 0.475, Figure 1P). Similarly, in wild-type animals, the number of surviving rod photoreceptor cells is equivalent (p = 0.0603) between morpholino treated (n = 22, average = 45.7) and control animals (n = 31, average = 40.1) at 108 hpf. These results suggest that the pathway of rod photoreceptor cell death passes mainly thought transducin.

### Rod photoreceptor cell death mechanism operates through adenylyl cyclase

**Figure 2:**
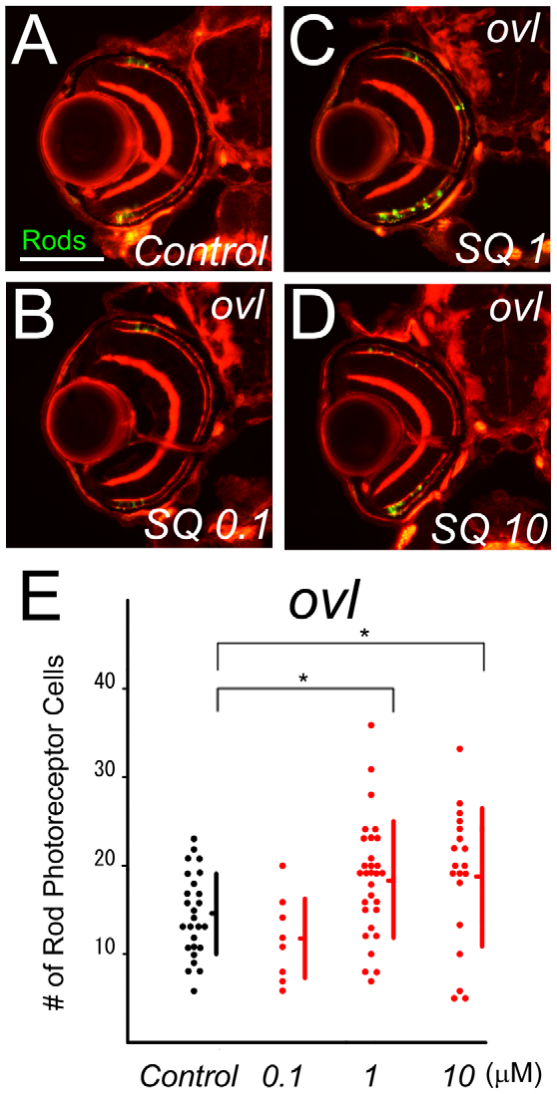
Inhibitor of ADCY suppresses photoreceptor cell death. (A–D) Sections of *ovl* mutants bred in SQ22536-treated water (B–D) and control water (A). (E) The number of surviving rod photoreceptors from *ovl* mutants in control water (black dots) and SQ22536-treated water. SQ22536 increased survival rod photoreceptors in concentrations of 1 and 10 mM. (Bars mean SD, * means p<0.05.)

**Figure 3:**
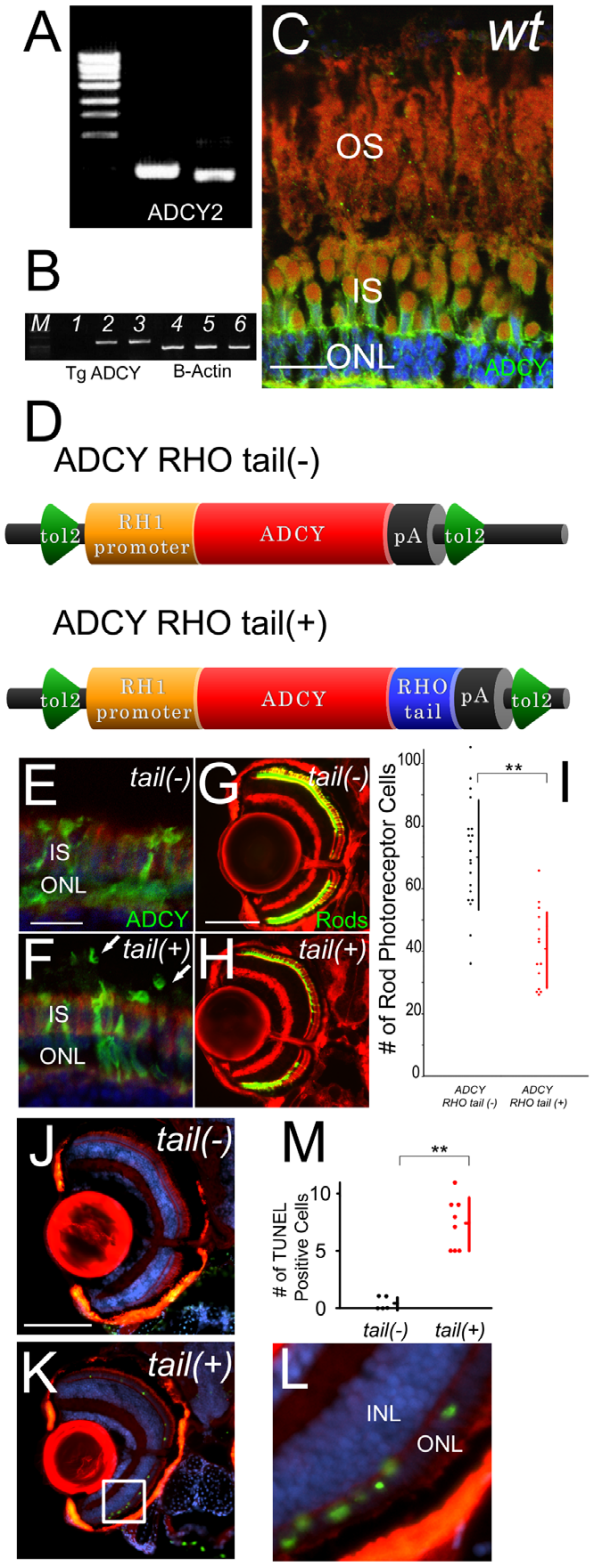
Mislocalized ADCY in rod outer segments induces photoreceptor cell death. (A) Expression analysis of adenylyl cyclases in wild-type retina by RT-PCR. (B) RT-PCR analysis of recombinant adenylyl cyclase 2B from wild-type (lane1), ADCY RHO tail (−) (lane2) and ADCY RHO tail (+) (lane3). Lanes 4 to 6 are B-actin expression of each group. Ectopic expressions were confirmed. (C) Immunohistochemistry (IHC) section of retina of wild-type. F-actin is visualized with phalloidin (red), ADCY2 with antibodies (green) and nuclei with Hoechst33342 (blue). OS: outer segment, IS: inner segment, ONL: outer nuclear layer (Bar = 10 µm.) ADCY did not expressed at OS. (D) Schematic diagrams of over-expression constructs. ADCY RHO tail (−) and (+) are downstream of zebrafish RH1 promoter between tol2 arms. (E and F) IHC sections of retina of ADCY RHO tail (−) fish (E) and ADCY RHO tail (+) fish (F) at 14 dpf. F-actin is visualized with phalloidin (red), ADCY2 with antibodies (green) and nuclei with Hoechst33342 (blue). Arrows indicate outer segments. IS: inner segment, ONL: outer nuclear layer (Bar = 10 µm.) ADCY is mis-localized at OS in only tail(+) animals. (G and H) Eye sections of ADCY RHO tail (−) and (+) animals at 14 dpf. Rod photoreceptors are visualized with EGFP (green) and F-actin with phalloidin (red). (Bar = 100 µm.) The number of rod photoreceptors was significantly decreased in tail (+) animals. (I) Graph of the number of rod photoreceptor of ADCY RHO tail (−) (black dots) and (+) (red dots). (Bars mean SD, ** means p<0.01.) (J and K) TUNEL (green) assay of sections in ADCY RHO tail (−) (J) and (+) (K) animals. F-actin is visualized with phalloidin (red), and nuclei with DAPI (blue). The signals of outer nuclear layer were observed only in tail (+) animals. (L) Magnification of the white square in (K). INL: inner uclear layer, ONL: outer nuclear layer. (M) Graph of the number of TUNEL assay positive cells, comparing ADCY RHO tail (−) (black dots) and (+) (red dots) animals. (Bars mean SD, ** means p<0.01.)

A sequential knockdown experiment suggests that the mislocalized phototransduction signal is connected to rod photoreceptor cell death signal through transducin, but how? Phototransduction is a typical G-protein coupled signaling pathway. Rhodopsin is a G-protein coupled receptor. Transducin is a Gt type GTP-binding protein, and PDE is its effecter. We hypothesized that an effecter molecule other than PDE mis-couples to transducin because of the ectopic expression of photopigments. Adenylyl cyclase (ADCY) is a candidate, because it is reported that ADCY 1, 2, and 4 are expressed in eye [18], [19] and we confirmed ADCY2 is expressed in the inner segments of photoreceptor cells, and is not expressed in the outer segments (Figure 3C). Alfinito et al. has proposed this mechanism using saramander photoreceptors *in vitro* [20]. To confirm this hypothesis, we applied both the agonist and antagonist of ADCY to *ovl*. SQ 22536, a cell-permeable adenylyl cyclase inhibitor, reduced rod photoreceptor cell death in *ovl* in a dose dependent manner. At 10 µM, SQ 22536 increased surviving rod photoreceptors in *ovl* retina by 38% (Figure 2A–E). On the other hand, in the wild-type retina, the ADCY inhibitor had no effect on photoreceptor survival. These results suggest that ADCY is an entry point to the rod photoreceptor cell death signaling pathway through phototransduction.

### Forced expression of adenylyl cyclase 2B in the outer segments leads to rod photoreceptor cell death

For further confirmation of the role of ADCY in rod photoreceptor cell death, we ectopically expressed ADCY in wild-type outer segments. Expression of ADCY [21]–[23] in the eye [19] has been reported. We first tested the expression of ADCY subtypes in eye. Among transmembrane ADCY subtypes, ADCY2B, 6, 8 and 9 were expressed in eye (result of RT-PCR of ADCYs is shown in Figure 3A), ADCY2 is also detected in eyes by western blotting (Figure S4) and immunohistological analysis showed that ADCY was expressed in photoreceptors and localized in the inner segments (Figure 3C) as reported [24]. To ectopically express ADCY in the outer segments, we used a fusion protein with rhodopsin C-terminus amino acids, because the rhodopsin C-terminus is essential for the apical projection of rhodopsin [25], [26], and the sequence had been used in some experiments for ectopic fusion protein projection to the outer segments [27]. We generated a transgenic ADCY2B fish with the rhodopsin C-terminus tail driven by zebrafish rhodopsin promoter, named “tail (+)”, and an ADCY2B fish without the rhodopsin tail named “tail (−)” (Figure 3D).

Expression of ADCY2B in ADCY transgenic tail (+) and tail (−) fish was confirmed by RT-PCR (Figure 3B). Sub-cellular localization of ectopically expressed ADCYs was confirmed by immunohistochemistry. In tail (+) fish, positive ADCY staining was detected in rod photoreceptor outer segments as well as in the inner segments by anti-adenylyl cyclase 2 antibody (Figure 3E, F). On the contrary, in tail (−) fish (Figure 3E), adenylyl cyclase was not detected in the outer segments, but was detected in the inner segments similar to wild-type expression (Figure 3C). We also confirmed that even in ADCY tail (+) fish, the transport of rhodopsin is not significantly affected (Figure S5). Thus, we were able successfully to express ADCY2B in the outer segments using the rhodopsin tail.

The number of rod photoreceptor cells in the adenylyl cyclase 2B tail (+) transgenic fish retina (n = 14, average = 41.0) was significantly reduced compared with that in the transgenic tail (−) fish retina (n = 20, average = 70.5) at 14 dpf (p<1.51E-8, Figure 3G–I).

The reduction of rod photoreceptor cell number in tail (+) fish was due to rod photoreceptor cell death, as apoptotic cell death was observed in only the tail (+) retina. The number of apoptotic photoreceptor cells in the adenylyl cyclase 2B tail (+) transgenic fish retina significantly increased (n = 8, average = 7.4), compared with that in transgenic rhodopsin tail (−) fish (n = 5, average = 0.40) at 14 dpf (p<1.36E-5, Figure 3J–M). Furthermore, as we expected, this rod photoreceptor cell death is light dependent. Mild light exposure increased the number of rod photoreceptors in ADCY tail (+) retina at 7 dpf (Figure S6, darkness group, average = 60.6; light group, average = 31.2; p = 4.27E-9). On the contrary, there is no significant difference in the number of cone photoreceptors between wild type and tail (+) fish (Figure S7, wild type, average = 97.3; tail (+), average = 101.8; p = 0.255). These results indicate activation of adenylyl cyclase coupled with mislocalization of phototransduction accelerates rod photoreceptor cell death.

### cAMP-related rod photoreceptor cell death

**Figure 4:**
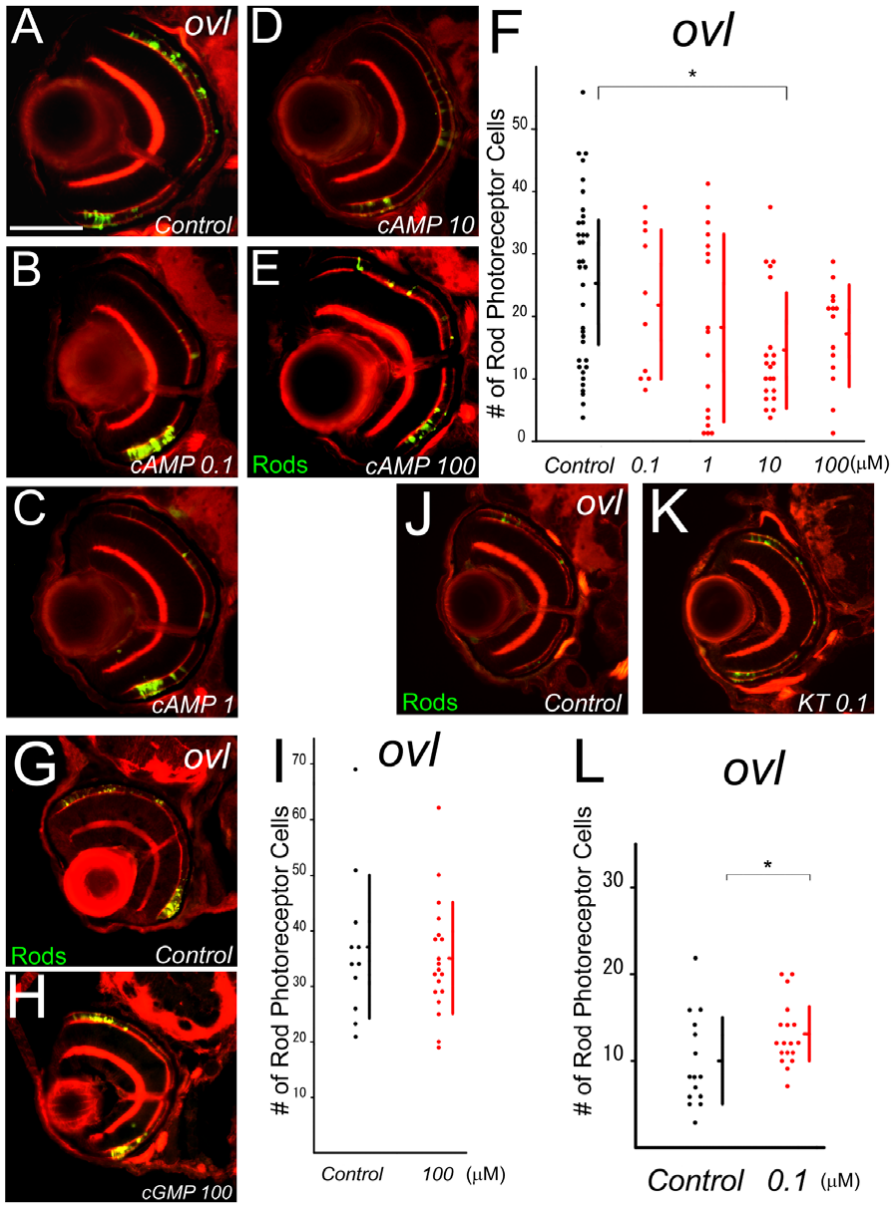
Treatment with cAMP analogue, cGMP analogue, and KT5720 in *ovl*. (A–E) Eye sections at 5 dpf *ovl* treated with different concentration of a cAMP analogue, 8-Bromo-cAMP. 8-Bromo-cAMP (B–E) or control water (A). Rod photoreceptors are visualized by EGFP (green) and F-actin by phalloidin (red). (Bar = 100 µm.) (F) Graph of survival rod photoreceptors of *ovl* mutants in control water (black dots) and cAMP analogue-treated water. cAMP analogue accelerated rod photoreceptor death. (Bars mean SD, * means p<0.05.) (G and H) Eye sections at 5 dpf *ovl* treated with an cGMP analogue, 8-Bromo-cGMP. (H) or control water (G). Rod photoreceptors are visualized with EGFP (green) and F-actin with phalloidin (red). (I) Graph of survival of *ovl* mutant rod photoreceptors in control water (black dots) and cGMP analogue-treated water. cGMP does not accelerate rod photoreceptor death. (Bars mean SD.) (J and K) Eye sections at 5 dpf *ovl* treated with KT5720 (K) or control water (J). Rod photoreceptors are visualized by EGFP (green) and F-actin by phalloidin (red). (L) Graph of survival of *ovl* mutant rod photoreceptors in control water (black dots) and KT5720 analogue-treated water. KT5720 suppresses rod photoreceptor death. (Bars mean SD, * means p<0.05.)

Activation of adenylyl cyclase generates cAMP at the inner segments of rods. Our next experiment investigated whether cAMP is related to rod cell death by using 8-Bromo-cAMP. 8-Bromo-cAMP is a cell permeable cAMP analog that we applied to *ovl* embryos. As we expected, the number of rod photoreceptor cells showed significant reduction (p<4.09E-5) between the 10 µM cAMP treated group (n = 22, average = 14.5) and a control group of *ovl* mutants (n = 36, average = 25.4) at 5 dpf (Figure 4A–F). Because the thickness of the inner nuclear layer (INL) was not affected significantly by cAMP treatment (Figure S8, control group, n = 5, average = 30.2 µm; cAMP treated group, n = 5, average = 29.8 µm; p = 0.298), so the decreased number of rod photoreceptors was not due to delay caused by the drug. On the other hand, 8-Bromo-cGMP did not show any effect on rod photoreceptor cell numbers in *ovl* mutants (p = 0.282; Figure 4G–I). These results show that the elevation of intracellular cAMP levels induces rod photoreceptor cell death.

PKA activation is an important downstream effect of cAMP induction. KT5720, an inhibitor of PKA, reduces rod photoreceptor cell death in *ovl*. At 0.1 µM, KT5720 increased surviving rod photoreceptors by 32% (p<0.0326) in the *ovl* retina (Figure 4J–L). These results indicate that cAMP plays a role in rod photoreceptor cell death. We also tested CREB phosphorylation, one of the main targets of PKA in *ovl* animals. We collected 80 eyes of *ovl* and *wt* animals and performed western blot by anti phosphorylated CREB (pCREB) and β-actin (We have tried blot of anti-CREB with two different antibodies, however, they did not work. So we used β-actin as a control). The pCREB level was increased 6.37% in ovl eye with normalized by b-actin blot level (Figure S9).

### Human rhodopsin mutant transgenic fish

**Figure 5:**
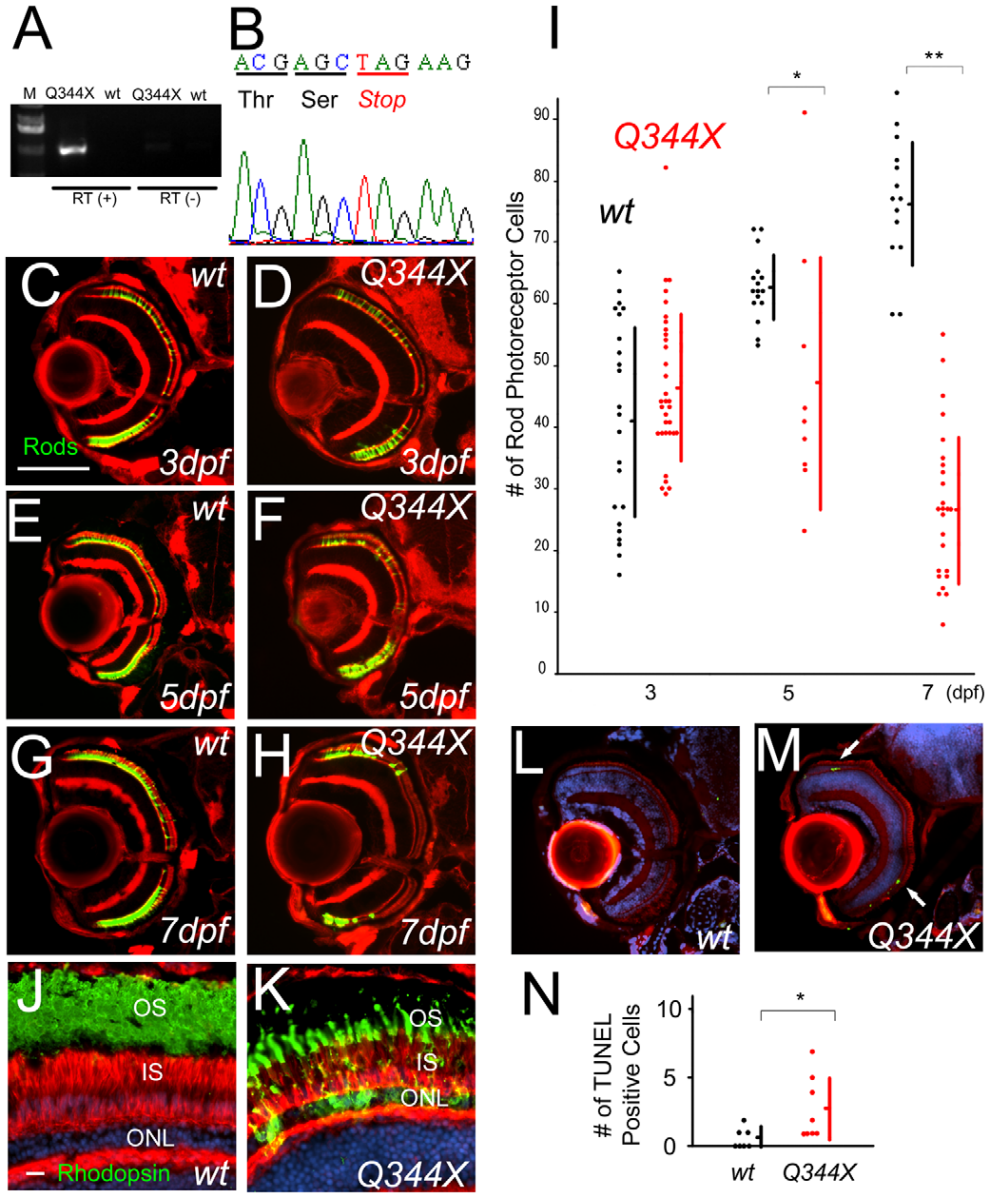
Rod photoreceptor cell death in rhodopsin Q344X transgenic fish. (A) RT-PCR analysis of expression of ectopic rhodopsin Q344X transgene. (B) Sequence analysis of transgene in Q344X animal at 5 dpf. (C–H) Sections of normal rhodopsin fish at 3 dpf (C), 5 dpf (E), 7 dpf (G) and rhodopsin Q344X transgenic fish at 3 dpf (D), 5 dpf (F), 7 dpf (H). Rod photoreceptors are visualized with EGFP (green) and F-actin with phalloidin (red). (Bar = 100 µm.) (I) Graph of the number of rod photoreceptor of normal rhodopsin and rhodopsin Q344X mutant at 3, 5 and 7 dpf. (Bars mean SD, * means p<0.05, ** means p<0.01.) Rod photoreceptors decreased by 5 dpf. (J and K) Immunohistochemistry sections of retina of wild-type (J) and Q344X (K) animal. F-actin is visualized with phalloidin (red), rod opsin with antibodies (green) and nuclei with Hoechst33342 (blue). OS: outer segment, IS: inner segment, ONL: outer nuclear layer (Bar = 10 µm.) Cell localization of rhodopsin is abnormal in Q344X. (L and M) TUNEL (green) assay of sections of normal rhodopsin (L) and rhodopsin Q344X transgenic (M) animals. F-actin is visualized with phalloidin (red), and nuclei with DAPI (blue). Arrows indicate TUNEL positive photoreceptor cells. TUNEL staining in ONL was observed only in Q344X. (N) Graph of the number of TUNEL assay positive cells, comparing normal rhodopsin (black dots) and rhodopsin Q344X (red dots) transgenic animals. (Bars mean SD, * means p<0.05.)

In *ovl*, ADCY plays a role in rod photoreceptor cell death via the phototransduction cascade. The essential questions are what is the photoreceptor cell death mechanism in *ovl*, and is it at work in human retinitis pigmentosa. To address these questions, we generated a transgenic fish with the human rhodopsin Q344X mutation. Q344X causes severe human RP and its transgenic rodents show similar photoreceptor degeneration [28]–[30]. As with the ADCY transgenic, we generated human rhodopsin-transgenic fish with and without the Q344X mutation, under the control of the zebrafish rhodopsin promoter. Because we induced human rhodopsin, we could confirm the ectopic expression of human rhodopsin in the transgenic fish by RT-PCR (Figure 5A). The transgenic fish expressed human rhodopsin only in the eye, whereas the wild-type did not express it at all. The Q344X mutation was confirmed by direct sequencing (Figure 5B). Immunohistochemistry revealed that in the Q344X transgenic fish, rhodopsin was not only detected in the outer segments but also in the entire cell membrane of the rod photoreceptor cell, as reported in mice and humans [30] (Figure 5J, K).

The number of rod photoreceptor cells was not significantly different in the wild-type human rhodopsin (no mutations) transgenic fish and rhodopsin Q344X mutation transgenic fish by 3 dpf (no mutations group; n = 23, average = 40.8, Q344X; n = 33, average = 46.5, p = 0.076, Figure 5C, D). However as the days passed, the Q344X retina showed photoreceptor degeneration. The number of rod photoreceptor cells in the Q344X retina at 5 (n = 9, average = 47.0, Figure 5F) and 7 dpf (n = 25, average = 27.4, Figure 5H) were significantly reduced compared with that in the unmutated rhodopsin transgenic fish at 5 (n = 16, average = 62.6, p<0.0280, Figure 5E) and 7 dpf (n = 14, average = 76.4, p<2.39E-14, Figure 5G, I). We confirmed by TUNEL assay that the degeneration in Q344X transgenic fish was due to apoptosis. The number of apoptotic photoreceptor cells in the rhodopsin Q344X transgenic fish retina (n = 8, average = 2.6) was significantly larger, compared with that in the wild-type retina (n = 8, average = 0.6) 5 dpf (p<0.0193, Figure 5L–N). Like *ovl* cones do not decrease in Q344X transgenic fish at 5 (p = 0.390), 7 dpf (p = 0.206) and even 1.5 mpf, (Figure S10, 11). These results demonstrate that the Q344X transgenic fish mimics human RP with the rhodopsin mutation.

**Figure 6:**
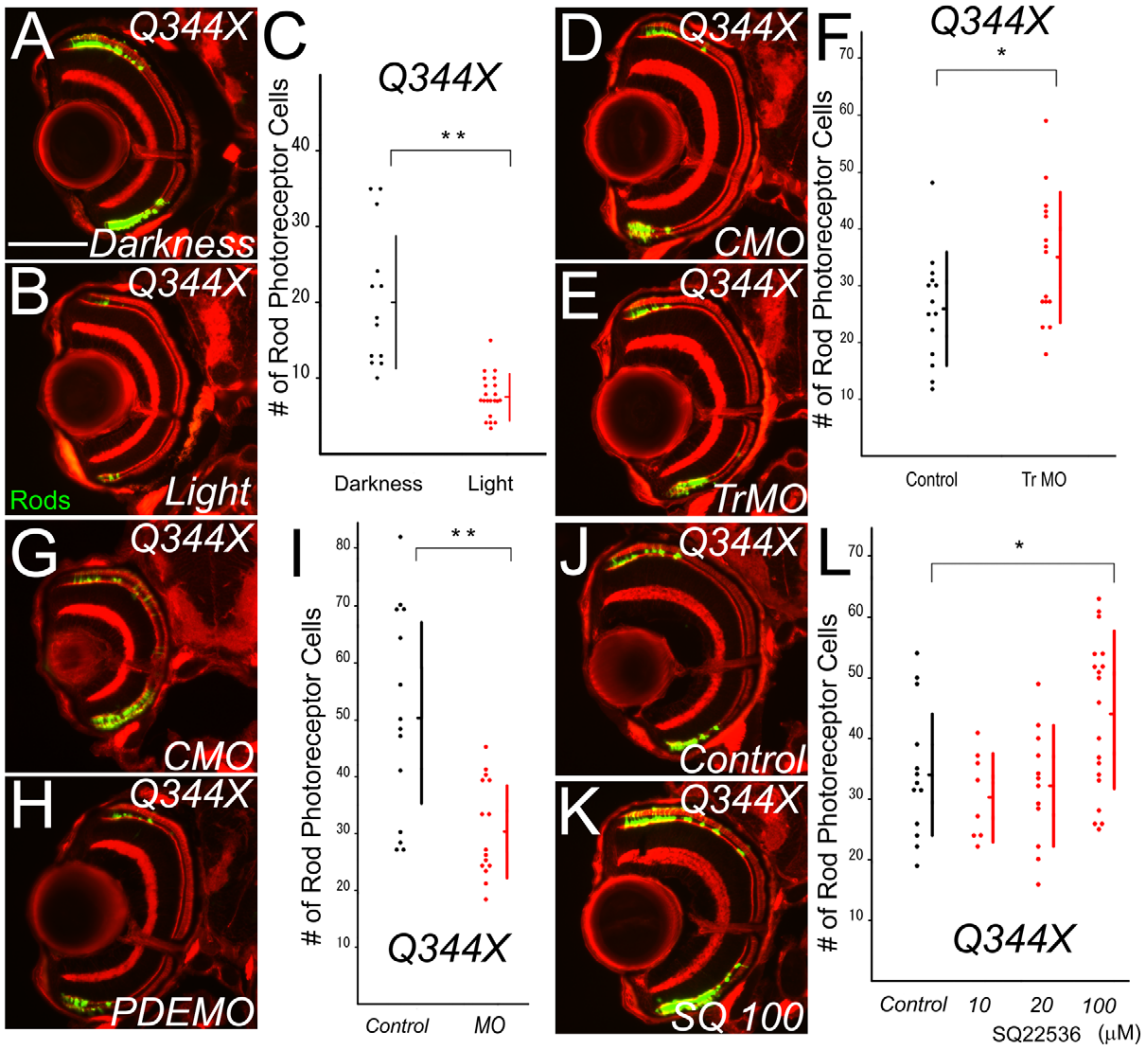
Effects on photoreceptor cell death in Q344X fish. (A and B) Retina sections of eyes from rhodopsin Q344X transgenic at 5 dpf. Animals were reared in constant darkness (A) or in constant light (B). Light exposure reduces the survival of rod photoreceptor cells. Rod photoreceptors are visualized by EGFP (Bar = 100 µm.) Light accelerated the rod cell death. (C) Graph of the number of rod photoreceptors in rhodopsin Q344X transgenic fish at 5 dpf. Darkness and light exposure are compared. (Bars mean SD, ** means p<0.01.) (D and E) Eye sections of eyes treated by anti-transducin morpholinos (E) and control MO (D) in Q344X at 5 dpf. Suppression of transducin α expression enhances the survival of rod photoreceptor cells. Rod photoreceptors are visualized by EGFP (Bar = 100 µm.). (F) Graph of the number of rods in Q344X, control morpholino-treated and anti-transducin morpholinos. (Bars mean SD, * means p<0.05.) (G and H) Eye sections of eyes treated by anti-phosphodiesterase 6β morpholinos (H) and control MO (G) in Q344X at 5 dpf. Suppression of phosphodiesterase expression reduces the survival of rod photoreceptor cells. Rod photoreceptors are visualized with EGFP (Bar = 100 µm.). (I) Graph of the number of rods in Q344X, control morpholino-treated and anti-phosphodiesterase 6β morpholinos. (Bars mean SD, ** means p<0.01.) (J and K) Eye ections of Q344X transgenic fish bred in SQ22536-treated water (K) and normal control water (J) at 5 dpf. Rod photoreceptors are visualized by EGFP (Bar = 100 µm.) ADCY antagonist rescued rod photoreceptor cell death. (L) Graph of the number of rod photoreceptor cells in Q344X 5 dpf. Black dots indicate control and red dots indicate SQ22536-treated (10, 20 and 100 mM) water. (Bars mean SD, * means p<0.05.)

Then, we tested the mechanism of rod photoreceptor cell death in the Q344X mutant. As shown in *ovl*, the number of rod photoreceptor cells showed a significant difference (p<1.13E-4) between the continuous light exposure (n = 21, average = 7.6) and control groups (n = 13, average = 20.5) in Q344X transgenic fish at 5 dpf (Figure 6A–C).

Morpholino knock down of transducin α significantly (p<0.0159) increased the number of surviving rod photoreceptor cells in the transducin morpholino-treated group (n = 15, average = 34.7) at 5 dpf, compared to the control morpholino-treated group (n = 14, average = 25.9) in the Q344X transgenic fish (Figure 6D–F). Over 33% of rods survived in transducin α morpholino-treated animals.

As in *ovl*, suppression of PDE6β did not rescue rod photoreceptor cell death in the Q344X transgenic. Injection of a PDE6β morpholino reduced the number of surviving rod photoreceptor cells in the Q344X mutant. The number of rod photoreceptor cells in the PDE6β morpholino-treated Q344X transgenic retina at 5 dpf was 30.5 (n = 15), whereas it was 50.6 (n = 14) in the control morpholino-treated Q344X transgenic retina (p = 7.9E-4; Figure 6G–I).

Rod photoreceptor cell death in the Q344X transgenic was also suppressed by ADCY antagonist in a dose dependent manner. Application of 100 µM of SQ22536 significantly (p<0.0179) increased the number of surviving rod photoreceptor cells (n = 19, average = 44.1) in the Q344X retina at 5 dpf, compared to controls (n = 13, average = 34.5). Concentrations of 10 and 20 µM of SQ 22536 did not significantly increase the number of rod photoreceptors (10 µM, n = 8, average = 30.5, p<0.164; 20 µM, n = 12, average = 31.8, p = 0.264), compared with controls (Figure 6J–L). These results suggest that ADCY plays a role in rod photoreceptor cell death in Q344X rhodopsin mutant, like *ovl*.

### ADCY suppression rescues photoreceptor cell death in mammalian RP model

**Figure 7:**
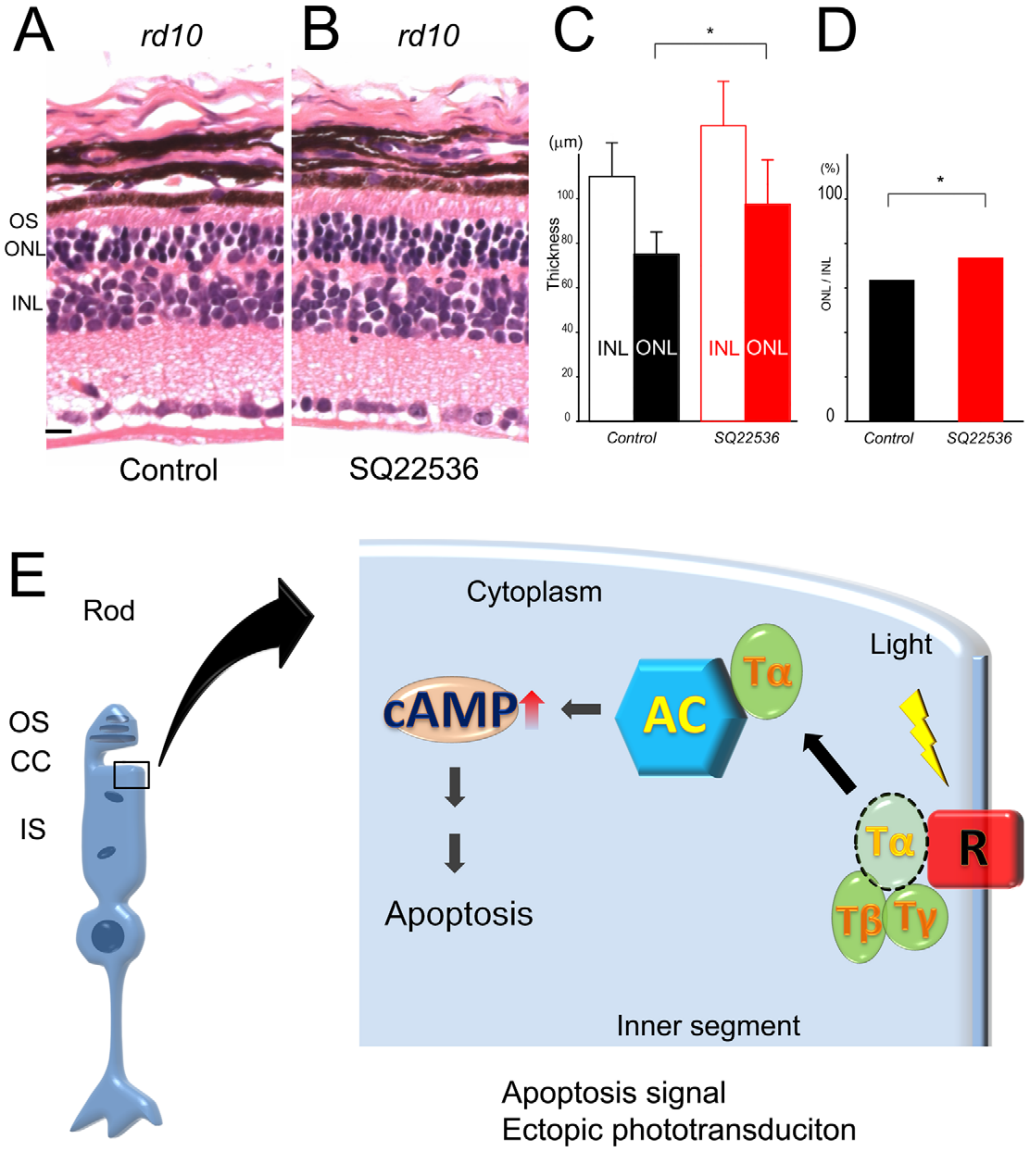
SQ22536 treatment of *rd10* mice. (A and B) HE (Hematoxilin-Eosin) stained sections from eyes of *rd10* mice at P28. Control PBS treated eye (A) and SQ22536 treated eye (B). OS: outer segment, IS: inner segment, ONL: outer nuclear layer (Bar = 10 µm.). (C) Graph of the thickness of INL (outlined bar) and ONL (solid bar) in *rd10* mice at P28. Control group (black) and SQ treated group (red) are compared. (Bars mean SD, * means p<0.05.) (D) Graph of the ONL/INL ratio of SQ22536 treated control (black bar) and untreated retina (red bar) in *rd10* mice. (E) Schematic illustration of adenylyl cyclase and apoptosis in rod photoreceptors. OS: outer segment, CC: connecting cilium, IS: inner segment, R: rhodopsin, T: transducin, AC: adenylyl cyclase.

The results of Q344X suggest that the ADCY plays a role in photoreceptor cell death in human RP. However, we still doubted that the phenomenon occurs only in fish. So we used a more common RP rodent model, *rd10* mice [31]. Mislocalized rhodopsin is also reported in *rd10* mice [31]. Mice homozygous for the *rd10* mutation show histological changes at postnatal day 16. *Rd10* mice have a point mutation in exon 13 of beta PDE [32], [33] and ectopic rhodopsin is found in the IS in photoreceptor cells. *Rd10* mice have less PDE protein than the wild-type in the retina [34]. We suspected that mislocalized phototransduction easily occurs in the *Rd10* retina, but actually, photoreceptor degeneration depends on light exposure [34]. We applied SQ22536, an adenylyl cyclase inhibitor, to *rd10* mice. A 10 µM/2 µL solution of SQ22536 was injected into the vitreous of one eye. We injected 2 µl PBS into the other eye as a control. The thickness of the retinal outer nuclear layer (ONL) was measured at 6 retinal points in each eye, and we calculate average of 6 points thicknesses. We injected SQ22536 or PBS at P18 and sectioned at P28. At 10 µM, SQ 22536 increased the ONL thickness by 29% in the *rd10* mice retina. The thickness of the ONL in the *rd10* mouse retina of the SQ22536 treated group significantly increased (n = 5, average = 16.2 µm, p<0.0314, Figure 7A), compared with the control group (n = 5, average = 12.6 µm, Figure 7B, C). However, it did not affect the thickness of the inner nuclear layer (INL) in the retina of the SQ22536 treated group (n = 5, average = 22.2 µm, p = 0.129), compared to the control group (n = 5, average = 19.9 µm). The ONL/INL ratio of the SQ22536 treated retina (0.729) increased compared to the control retina (0.630) in *rd10* mice (Figure 7D). The results using *ovl*, Q344X transgenic, and *rd10* mice indicate that adenylyl cyclase may be commonly involved the photoreceptor cell death pathway in human retinitis pigmentosa.

## Discussion

In the eye, the photoreceptor is the light detector, and the relationship between light and photoreceptor death has been discussed [35]–[37]. RP is a slow progressive disease and most patients are concerned about light exposure accelerating the progression of the disease.

Hao et al. [38] revealed that, in a mutant mouse model, phototransduction has an apparent relationship to photoreceptor cell death. They also reported that photoreceptor cell death is caused by even relatively low-level light exposure, which is not harmful for wild-type photoreceptors. The cell death mechanism is different from that caused by strong light exposure in the RP retina, but the details are not yet known.

In photoreceptor cell death in rhodopsin mutant, some investigators suggest that transducin is involved [39], and the others suggest not [40]. Our results of genetic manipulation and antagonist experiments prefer the former hypothesis in rod photoreceptor cell death caused by relatively low-level light exposure. In our experiment, PDE does not affect the rod photoreceptor cell death in *ovl*. However, it is possible that the knockdown of PDE causes rod generation in mammals. So we could not conclude that PDE is not involved in photoreceptor cell death caused by light, however, our results suggest that transducin is included in rod photoreceptor cell death caused by light.

Alfinito [20] first proposed that ADCY is related to photoreceptor cell death, however, they used digested and unhealthy salamander photoreceptors *in vitro*, and only used antagonists in their experiments, so it had been still unknown whether the same holds true for human RP. In this report we used several lines of investigation that shows ADCY is involved in rod photoreceptor cell death. This adenylyl cyclase mechanism is seen not only in *ovl*, but also in the human rhodopsin mutation transgenic Q344X, and a mouse model. So it might be shown in human RP.

Cyclic AMP is a major second messenger with various biological effects including apoptosis, which it can in some cases induce and in others reduce. Some reports demonstrated that in diffuse large B-cell lymphoma cells, cAMP induces cell death via the phosphatidylinositol 3-kinase (PI3K)/AKT pathway [41], and in WT S49 T-lymphoma cells, cAMP induces cell death via Bim [42]. The pathway from cAMP to PKA is also known as a cell death factor in coronary endothelial cells via mitochondrial Bax and caspase3 [43]. In the retina of rhodopsin mutant transgenic mice, elevation of the cAMP level was reported [44]. In our results, cell permeable cAMP, not cGMP, could induce rod photoreceptor cell death. These results strongly suggest that elevation cAMP seems to play an important role in some rod photoreceptor cell death pathways. We also found that PKA inhibitor KT5720 partially rescued rod photoreceptor cell death in *ovl*, and the level of pCREB is increased in *ovl* eye. However, we are still wondering that the role of PKA, especially CREB phosphorylation level in rod photoreceptor degeneration. First, total CREB level is unknown, because the antibodies did not work in zebrafish. So it is possible that if the CREB translation level itself is increased, the pCREB level is also increased without PKA activation. Second, the down-regulation of pCREB level in photoreceptor degeneration is reported. In *rds* mice retina cAMP level is abnormally high [45], but CREB activation and expression is downregulated [46]. Interestingly, in *rd1* not only PKA but also other CREB kinases (Akt [47], PKC [48], CaMKII [49], ERK1/2 [50]) are activated. Increased activity of CREB kinases in spite of downregulation of CREB activity might indicate that CREB activity might be strongly suppressed under the kinases activation. Inducible cAMP early repressor (ICER) is thought to be a candidate modifier, because it works as endogenous CREB antagonist, which might be dependent on PKA activity [51]. The role of PKA and CREB in photoreceptor cell death should be examined further more.

Here, we demonstrated a mechanism of rod photoreceptor cell death caused by light *in vivo* (Figure 7E). In this mechanism, ectopic phototransduction is connected to ADCY via transducin leading rod photoreceptor cell death. It was observed in zebrafish mutants, human rhodopsin mutant transgenic fish, and *rd10* mice. However, RP is a group of diseases, and response to light exposure is quite different among models. For example, our mammalian model, *rd10* is the pde6e mutant mouse. The mutation can cause photoreceptor cell death but the mechanism might be independent to ADCY pathway we have demonstrated. This is the reason why photoreceptor rescue is weak in *rd10* mice by shutdown of ADCY pathway. On the contrary, even in *ovl*, ADCY pathway is not only the cause of photoreceptor cell death, because complete darkness treatment could not show the perfect rescue. So, the cell death mechanism presented here cannot explain all mechanisms of photoreceptor cell death. It is possible that the mechanism is not a main pathway in some patients. However, we still think it is an important mechanism. Because RP is a late onset and slowly progressive disease, patients usually have no obvious symptoms in childhood with no complains. In our research, the average age when patients were diagnosed with RP is 35 years old [52], and visual loss progresses very slowly leading to blindness. So prevention of any pathway of rod photoreceptor cell death would be a great help in maintaining a patient's quality of life. However, to date, there are no effective cell protection therapies. The cell death pathway that we have demonstrated includes novel target molecules, adenylyl cyclase and PKA, and may be helpful in developing an effective drug treatment for RP.

## Materials and Methods

### Fish Strains

The maintenance and breeding of zebrafish strains and staging of embryonic development were performed as described [53], [54]. The *ovl* allele, originally recovered in a large-scale mutagenesis screen [55], was initially characterized in a previous study [56]. RH1upEGFP fish is a gift from Dr. Kawamura [57]. In this line, rod photoreceptors were visualized with EGFP driven by zebrafish rhodopsin promoter (RH1).

### *Rd10* mice

The P18 inbred *rd10* mice were maintained humanely, with proper institutional approval, and in accordance with the ARVO Statement for the Use of Animals in Ophthalmic and Vision Research. All animal experiments were carried out under approved protocols and in accordance with the recommendations for the proper care and use of laboratory animals by the Committee for Animals, and Infectious Pathogens Experiments at Kyushu University (Approval ID: A22-070-0) and according to The Law (No. 105) and Notification (No. 6) of the Japanese Government. In the sections below, n is used to indicate the number of treated eyes.

### Molecular biology (PCR and RT-PCR)

PCR was carried out in a 50 µl reaction mixture containing 100 ng of genomic DNA, 50 pmol of each primer, 2.0 mM MgCl2, 1× reaction buffer (Takara), 200 µM of each dNTP, and 1.0 U of Ex Taq® polymerase (Takara). Samples were amplified for 30 cycles, 30 s at 94°C, 30 s at 60°C, and 30 s at 72°C.

Total RNA was extracted from embryos using ISOGEN (Wako) and prepared for cDNA synthesis using SuperScript®II transcriptase (Invitrogen). Reverse transcription was performed in a 20 µl reaction volume, containing ∼1 µg total RNA, 500 ng Oligo(dT)12–18, 500 µM of each dNTP, 10 mM DTT, and 50 units of SuperScript®II (Invitrogen) at 42°C for 50 min. PCR conditions were essentially the same as in the genomic PCR described above except the annealing temperature was changed from 58°C to 62°C, and the extension time was changed from 30 s to 5 min. PCR was performed with gene-specific primers. PCR products were purified using the QIAquick® PCR Purification Kit (Qiagen) following the user manual and sequenced. Mutations were detected by direct sequencing. The primers used are as follows: β-Actin, 5′-TGGTATTGTGATGGACTCTGG-3′ and 5′-AGCACTGTGTTGGCATACAGG-3′; Transducin α, 5′-ATCAAAAGTCAGTATGGGGGCCGG-3′ and 5′-CTGTGCGGCAGCGTCTCCATAG-3′; PDE6β, 5′-CTCCAGACTCTGAGATCGTC-3′ and 5′-CACAGTTGTGAAGGTAGCTC-3′; ADCY2B(1), 5′-CGCCTTGATCCTCTGCATTTGTTT-3′ and 5′-CCCGGCCGGTCTGGTTG-3′; ADCY2B(2), 5′-TGGCGAGGCAGAATGAATA-3′ and 5′-TACTGCCGGTCGTGATACTG-3′; Transgenic ADCY2B tail(−)(+), 5′-ATAAAGAGGGGTTGGAGTGT-3′ and 5′-TGTGGTATGGCTGATTATGATC-3′; Transgenic rhodopsin Q344X, 5′-CCAGCGTGGCATTCTACATC-3′ and 5′-AACGCTTACAATTTACGCCT-3′.

### Morpholino Knock Down

Morpholino oligonucleotide knock downs were performed as described previously [54]. The concentration of MOs used in the experiments is 380 µM. For each gene, we used a morpholino targeted to a splice site of the genes (SP morpholinos). These morpholinos block the splicing and the efficiency of SP morpholino knock down was determined by RT-PCR analysis. The following morpholinos were used: Transducin α (SP1), 5′-CAGCACCTGAAAGCAAGACAGTGTT-3′; Transducin α (SP2), 5′-TCTGGGATGGAGAAAGATACGTTTA-3′ (GENE TOOLS, LLC); PDE6β (SP), 5′-GACTGTCCTACAGCGAAAACAAAGT-3′ (GENE TOOLS, LLC); and Standard Control Morpholino, 5′-CCTCTTACCTCAGTTACAATTTATA-3′ (GENE TOOLS, LLC).

### Histology

For histological analysis of zebrafish, embryos were fixed in 4% paraformaldehyde (PFA, w/v, pH 7.4) in PBST overnight at 4°C. Embedding, sections (with 16 µm thickness along the lens/optic nerve axis, only one section from only one side of eyes per embryo), and staining were performed as described previously [58], [59]. So Ns appeared in text and figures mean the number of animals. The phenotype of the embryos was observed using a Zeiss Axioscope microscope or Bio-Rad Confocal Microscopy Radiance 2100 system. Images were recorded using digital cameras and processed with AxioVision (Carl Zeiss), LaserSharp2000 (Carl Zeiss) or Adobe Photoshop software (Adobe, Inc.). The number of rod photoreceptor cells was counted as fluorescent positive cells in each photo.

### Transgenic Animals

Human rhodopsin Q344X transgenic fish:

The *tol2* transposon system was used to produce transgenic zebrafish with the rhodopsin Q344X mutation associated with autosomal dominant Retinitis Pigmentosa in humans.

ADCY 2 transgenic fish with C-terminal of rhodopsin (RHO tail (+)) and without C-terminal of rhodopsin (RHO tail (−)):

Using the *tol2* transposon system, we produced transgenic zebrafish with the adenylyl cyclase 2 RHO tail (+) or RHO tail (−). Constructs consist of a fusion protein of ADCY2B and rhodopsin C-terminus tail 38 amino acids (KQFRNCMLTTICCGKNPLGDDEASATVSKTETSQVAPA, rhodopsin tail) driven by the zebrafish rhodopsin promoter.

### Drug, Reagent, and Medication

Fish were bred in water with the following drugs from 60 hpf to 108 hpf:

SQ22536 (Product Number: S153, Sigma-Aldrich Co.), 8-Bromo-cAMP sodium salt (Cat. No: 1140, Tocris Bioscience.), 8-Bromoguanosine 3′, 5′-cyclic monophosphate sodium salt (Product Number: B1381, Sigma-Aldrich Co.), and KT5720 (Cat. No: 1288, Tocris Bioscience.) Side effects of 8-Bromo-cAMP were quantified by the thickness of INL which was measured at three points (center and 1/4 distances from the ora serrata and the optic nerve head) in each of the nasal and temporal hemispheres of the eye.

### Immunohistochemistry and Immunoblotting

Antibody staining was performed on whole animals or on frozen sections as described in previous publications [58], [59]. The following primary antibodies and dilutions were used: mouse anti-rhodopsin (1∶5000, ABR), mouse zpr-1 (1∶200), rabbit anti-CREB (1∶500, abcam), rabbit anti-pCREB (phosphor S133: 1∶500, abcam) and mouse anti-adenylyl cyclase 2 (1∶200, Abnova). To visualize F-actin, rhodamine-conjugated phalloidin (1∶250, Sigma) was added to the secondary antibody solution. Confirmation of the expression for ADCY2, B-actin and pCREB was obtained by western blot analysis (NuPAGE electrophoresis system, iBlot Dry Blotting System, Invitrogen).

### Evaluation of Light Damage

Embryos, in light exposure experiments (Figure 1H, 6C and S6), were reared on a light cycle of 14 hr of light exposure (300 Lux) and 10 hr of darkness (0 Lux) at 28°C until 72 hpf and subsequently subdivided into 2 groups that were treated with the following light exposure regimen until 120 hpf: constant light of 800 Lux or constant darkness (0 Lux). Other experiments were done in light cycle condition. Embryos were collected at appropriate time points, fixed, cryosectioned, and stained with anti-rod opsin and adenylyl cyclase 2 antibodies as above. Light intensity was measured using Model LX-1108 digital light meter (Lutron). A nonpaired t test was performed to determine the statistical significance of cell number changes.

### Apoptosis assays

TUNEL (TdT-mediated dUTP Nick-End Labeling) assay: Apoptotic cell death was detected using the ApopTag® Fluorescein In Situ Apoptosis Detection Kit (Millipore) standard protocols.

### Other Protocols

Retinal sections of *rd10* mice

The mice were sacrificed at P18, and the eyes were enucleated and fixed with ice-cold 4% paraformaldehyde in PBS. Twenty-four hours later, the samples were embedded in paraffin, and 5 µm thick sections along the pupil/optic nerve axis were examined under a light microscope. Neuroprotective effects were quantified by the thickness of ONL which was measured at three points (center and 1/4 distances from the ora serrata and the optic nerve head) in each of the nasal and temporal hemispheres of the eye. The sections were observed using OLYMPUS BX50 microscope system. Images were recorded using digital cameras and processed with AxioVision (Carl Zeiss) or Adobe Photoshop software (Adobe, Inc.).

Genbank accession numbers

Rhodopsin (human): NM_000539, Transducin α (zebrafish): NM_131868, pde6b (zebrafish): XM_679910, ADCY2 (zebrafish): NM_001099987

## Supporting Information

Figure S1 **Rod photoreceptor cell death in** ***ovl*** **.** (A–F) Sections of wild type fish at 3 (A), 5 (C) 7 dpf (E) and *ovl* at 3(B), 5(D) 7 dpf (F). Rod photoreceptors were visualized with EGFP (green) and F-actin with phalloidin (red). (Bar = 100 µm.) (G) The number of rod photoreceptor of wild type fish and *ovl* during development (Bars mean SD, ** means p<0.01.).
(DOC)

Figure S2 **Cones are not decreased in** ***ovl*** **at 4 dpf.** (A and B) Sections of eyes from wt (A) and *ovl* (B) fish at 108 hpf. R/G cone photoreceptors were visualized with zpr1 (red), rod photoreceptors are visualized with EGFP (green) and nuclei with Hoechst33342 (blue). (Bar = 100 µm.) There were no significant changes. (C) The number of R/G cone photoreceptors in wt (black dots) and *ovl* fish (red dots) at 4 dpf. wild type, average = 72.6; *ovl*, average = 69.1; p = 0.235. Bars mean SD.
(DOC)

Figure S3 **Transducin morpholino suppresses photoreceptor apoptosis in** ***ovl*** **.** (A and B) TUNEL (green) assay of sections of control (A) and transducin morpholino treated (B) in *ovl*. F-actin is visualized with phalloidin (red), and nuclei with DAPI (blue). Arrow heads indicate TUNEL positive cells in outer-nuclear layer in control animals. (N) The number of TUNEL assay positive cells in outer-nuclear layer, comparing control (black dots) and transducin morpholino treated (red dots) in *ovl*. control group, average = 10.6; transducin α morpholino treated group, average = 1.8; p = 0.00136. Bars mean SD, ** means p<0.01.).
(DOC)

Figure S4 **Western blotting of the** ***ovl*** **fish with ADCY 2 antibody.** Arrow head indicate specific expected band (38.2 kDa).
(DOC)

Figure S5 **Transport of rhodopsin is not significantly affected in ADCY RHO tail (+) fish.** (A) Rhodopsin staining on the sections of retina of ADCY RHO tail (+) fish. (B) Magnification of white square in (A). Rhodopsin was normally transported to outer segments. F-actin is visualized with phalloidin (red) and rhodopsin with antibodies (green). (Bar = 100 µm.)
(DOC)

Figure S6 **Rod photoreceptor cell death in ADCY RHO tail (+) is light dependent.** (A and B) Animals were reared in constant darkness (A) or in constant light (B). Light exposure reduces the survival of rod photoreceptor cells. Rhodopsin is visualized by antibody (green) and F-actin by phalloidin (red). (Bar = 100 µm.) (C) The number of survived rod photoreceptors in ADCY RHO tail (+) fish under constant darkness (black dots) and under constant light (red dots). (Bars mean SD, * means p<0.05.)
(DOC)

Figure S7 **Cones are not decreased in ADCY RHO tail (+) fish.** (A and B) Sections of eyes from wt (A) and ADCY RHO tail (+) fish (B) at 7 dpf. R/G cone photoreceptors were visualized with zpr1 (green) and F-actin by phalloidin (red). (Bar = 100 µm) No significant difference was observed under the normal light condition. (C) The number of R/G cone photoreceptors in wt (black dots) and ADCY RHO tail (+) fish (red dots) at 7 dpf. (Bars mean SD.)
(DOC)

Figure S8 **Inner nuclear layer is not affected by 8-Bromo-cAMP treatment.** (A and B) Eye sections at 5 dpf *ovl* treated with 10 µM of a cAMP analogue, 8-Bromo-cAMP (B) and control (A). There are no significant differences. (C) The thickness of inner nuclear layer was not affected by 8-Bromo-cAMP treatment.
(DOC)

Figure S9 **Western blotting of wild type and the** ***ovl*** **fish with pCREB antibody.** Western blot of phosphorylated CREB (A) and β-actin (B) in ovl eye. Anti-CREB antibodies did not work in zebrafish, so we used β-actin as a control. The numbers below the blot is the raw densitometry data. pCREB is increased 6.37% under the normalization by β-actin.
(DOC)

Figure S10 **Cones are not decreased in rhodopsin Q344X transgenic fish.** (A and B) Sections of eyes from wt (A) and Q344X transgenic fish (B) at 5 dpf. R/G cone photoreceptors were visualized with zpr1 (green) and F-actin by phalloidin (red). (Bar = 100 µm.) There were no significant changes. (C) The number of R/G cone photoreceptors in wt (black dots) and Q344X transgenic fish (red dots) at 5 dpf. (Bars mean SD.) (D and E) Sections of eyes from wt (D) and Q344X transgenic fish (E) at 7 dpf. R/G cone photoreceptors were visualized with zpr1 (green) and F-actin by phalloidin (red). (C) The number of R/G cone photoreceptors in wt (black dots) and Q344X transgenic fish (red dots) at 7 dpf. (Bars mean SD.)
(DOC)

Figure S11 **Cones do not significantly degenerate in rhodopsin Q344X transgenic fish at 1.5 mpf.** (A and B) Sections of eyes from wt (A) and Q344X transgenic fish (B) at 1.5 mpf. UV cone photoreceptors were visualized with EGFP (green) and rhodopsin by antibody (red). OS: outer segment, IS: inner segment.
(DOC)
