# Supplementary material for: The Role of Mislocalized Phototransduction in Photoreceptor Cell Death of Retinitis Pigmentosa
Source: PLoS One. 2012 Apr 2;7(4):e32472. doi: 10.1371/journal.pone.0032472 (PMC3317642; doi:10.1371/journal.pone.0032472)
Supplement: Figure S1 — Rod photoreceptor cell death in ovl . (A–F) Sections of wild type fish at 3 (A), 5 (C) 7 dpf (E) and ovl at 3(B), 5(D) 7 dpf (F). Rod photoreceptors were visualized with EGFP (green) and F-actin with phalloidin (red). (Bar = 100 µm.) (G) The number of rod photoreceptor of wild type fish and ovl during development (Bars mean SD, ** means p<0.01.). (DOC) [file pone.0032472.s001.doc]

Figure S1. Rod photoreceptor cell death in *ovl*.


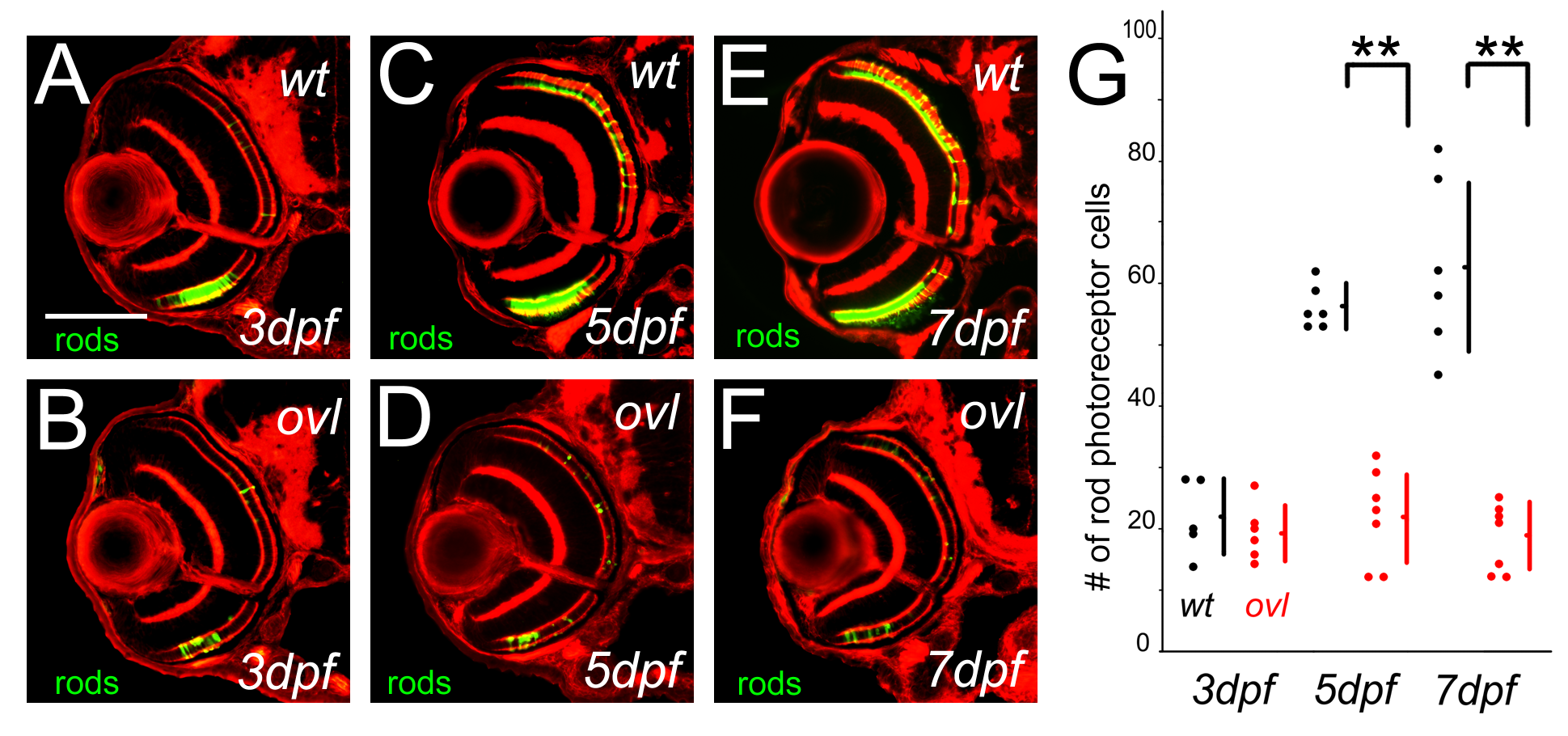


(A-F) Sections of wild type fish at 3 (A), 5 (C) 7 dpf (E) and *ovl* at 3(B), 5(D) 7 dpf (F). Rod photoreceptors were visualized with EGFP (green) and F-actin with phalloidin (red). (Bar = 100 µm.)

(G) The number of rod photoreceptor of wild type fish and *ovl* during development. (Bars mean SD, ** means p < 0.01.)
