# Supplementary material for: The Role of Mislocalized Phototransduction in Photoreceptor Cell Death of Retinitis Pigmentosa
Source: PLoS One. 2012 Apr 2;7(4):e32472. doi: 10.1371/journal.pone.0032472 (PMC3317642; doi:10.1371/journal.pone.0032472)
Supplement: Figure S2 — Cones are not decreased in ovl at 4 dpf. (A and B) Sections of eyes from wt (A) and ovl (B) fish at 108 hpf. R/G cone photoreceptors were visualized with zpr1 (red), rod photoreceptors are visualized with EGFP (green) and nuclei with Hoechst33342 (blue). (Bar = 100 µm.) There were no significant changes. (C) The number of R/G cone photoreceptors in wt (black dots) and ovl fish (red dots) at 4 dpf. wild type, average = 72.6; ovl, average = 69.1; p = 0.235. Bars mean SD. (DOC) [file pone.0032472.s002.doc]

Figure S2. Cones are not decreased in *ovl* at 4 dpf.

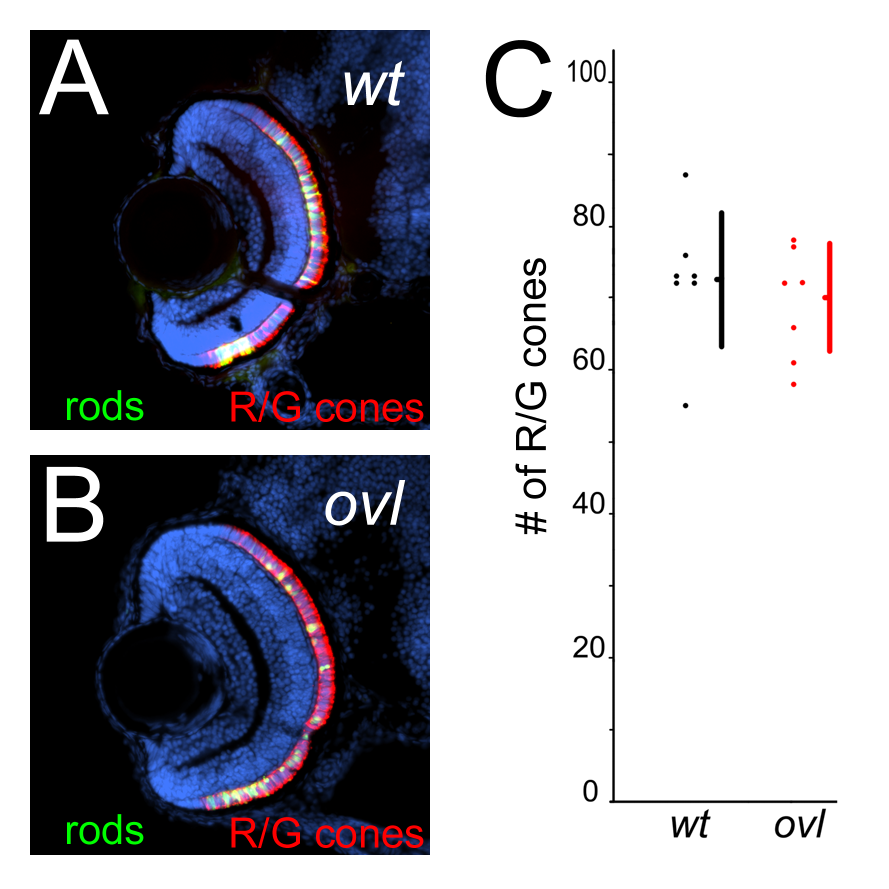


(A and B) Sections of eyes from wt (A) and *ovl* (B) fish at 108 hpf. R/G cone photoreceptors were visualized with zpr1 (red), rod photoreceptors are visualized with EGFP (green) and nuclei with Hoechst33342 (blue). (Bar = 100 µm.) There were no significant changes.

(C) The number of R/G cone photoreceptors in wt (black dots) and *ovl* fish (red dots) at 4 dpf. (wild type, average = 72.6; *ovl*, average = 69.1; p = 0.235. Bars mean SD.)
