# Supplementary material for: The Role of Mislocalized Phototransduction in Photoreceptor Cell Death of Retinitis Pigmentosa
Source: PLoS One. 2012 Apr 2;7(4):e32472. doi: 10.1371/journal.pone.0032472 (PMC3317642; doi:10.1371/journal.pone.0032472)
Supplement: Figure S3 — Transducin morpholino suppresses photoreceptor apoptosis in ovl . (A and B) TUNEL (green) assay of sections of control (A) and transducin morpholino treated (B) in ovl. F-actin is visualized with phalloidin (red), and nuclei with DAPI (blue). Arrow heads indicate TUNEL positive cells in outer-nuclear layer in control animals. (N) The number of TUNEL assay positive cells in outer-nuclear layer, comparing control (black dots) and transducin morpholino treated (red dots) in ovl. control group, average = 10.6; transducin α morpholino treated group, average = 1.8; p = 0.00136. Bars mean SD, ** means p<0.01.). (DOC) [file pone.0032472.s003.doc]

Figure S3. Transducin morpholino suppresses photoreceptor apoptosis in *ovl*.


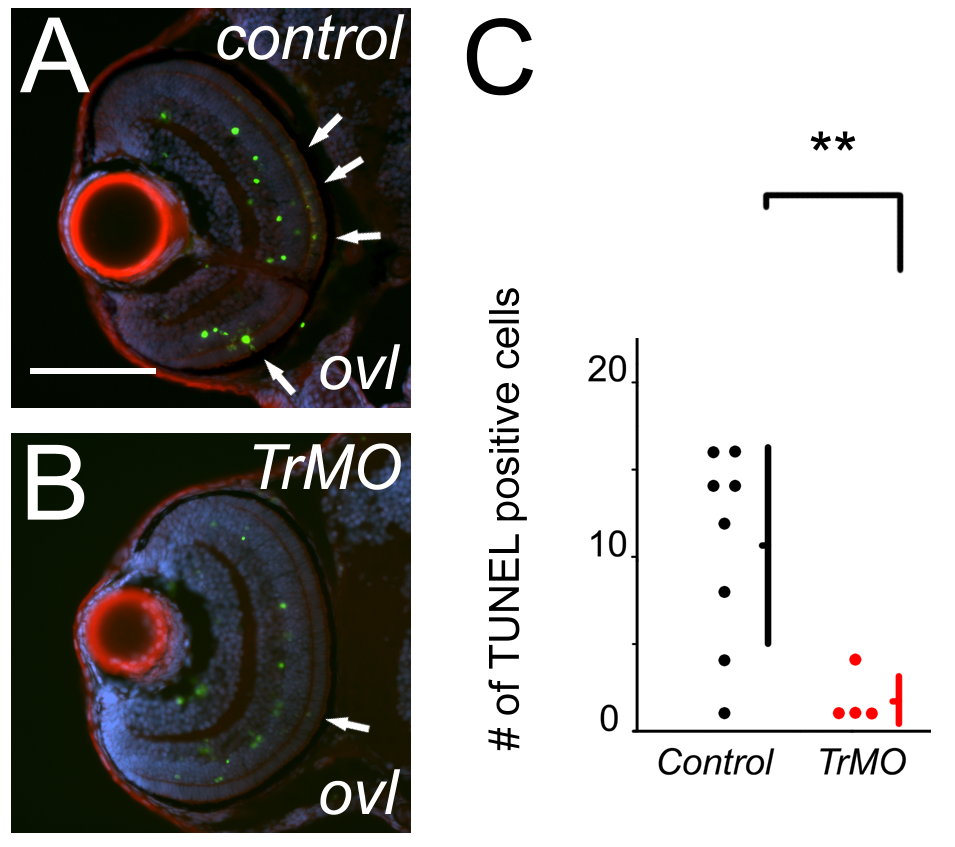


(A and B) TUNEL (green) assay of sections of control (A) and transducin morpholino treated (B) in *ovl*. F-actin is visualized with phalloidin (red), and nuclei with DAPI (blue). Arrow heads indicate TUNEL positive cells in outer-nuclear layer in control animals.

(C) The number of TUNEL assay positive cells in outer-nuclear layer, comparing control (black dots) and transducin morpholino treated (red dots) in *ovl*. (control group, average = 10.6; transducin α morpholino treated group, average = 1.8; p= 0.00136; Bars mean SD, ** means p < 0.01.)
