# Supplementary material for: The Role of Mislocalized Phototransduction in Photoreceptor Cell Death of Retinitis Pigmentosa
Source: PLoS One. 2012 Apr 2;7(4):e32472. doi: 10.1371/journal.pone.0032472 (PMC3317642; doi:10.1371/journal.pone.0032472)
Supplement: Figure S4 — Western blotting of the ovl fish with ADCY 2 antibody. Arrow head indicate specific expected band (38.2 kDa). (DOC) [file pone.0032472.s004.doc]

Figure S4. Western blotting of the *ovl* fish with ADCY 2 antibody.


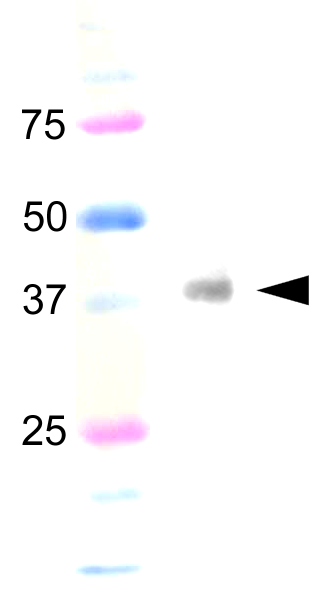


Arrow head indicate specific expected band (38.2 kDa).
