# Supplementary material for: The Role of Mislocalized Phototransduction in Photoreceptor Cell Death of Retinitis Pigmentosa
Source: PLoS One. 2012 Apr 2;7(4):e32472. doi: 10.1371/journal.pone.0032472 (PMC3317642; doi:10.1371/journal.pone.0032472)
Supplement: Figure S5 — Transport of rhodopsin is not significantly affected in ADCY RHO tail (+) fish. (A) Rhodopsin staining on the sections of retina of ADCY RHO tail (+) fish. (B) Magnification of white square in (A). Rhodopsin was normally transported to outer segments. F-actin is visualized with phalloidin (red) and rhodopsin with antibodies (green). (Bar = 100 µm.) (DOC) [file pone.0032472.s005.doc]

Figure S5. Transport of rhodopsin is not significantly affected in ADCY RHO tail (+) fish..


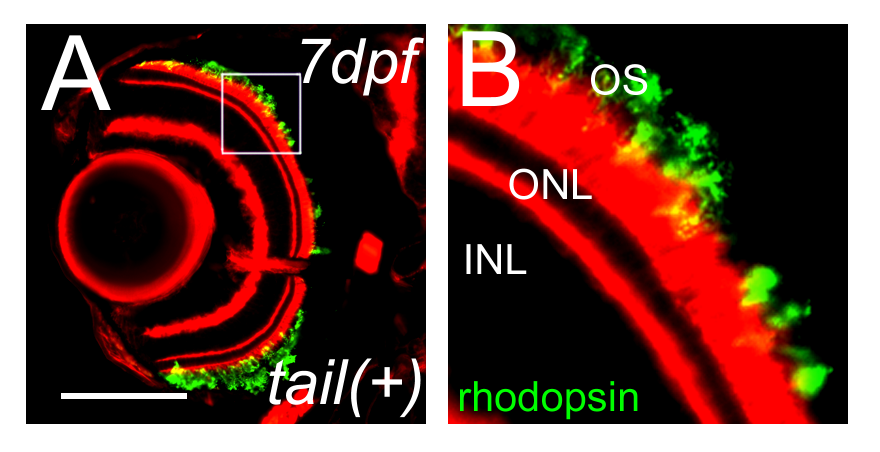


(A) Rhodopsin staining on the sections of retina of ADCY RHO tail (+) fish.

(B) Magnification of white square in (A). Rhodopsin was normally transported to outer segments. F-actin is visualized with phalloidin (red) and rhodopsin with antibodies (green). (Bar = 100 µm.)
