# Supplementary material for: The Role of Mislocalized Phototransduction in Photoreceptor Cell Death of Retinitis Pigmentosa
Source: PLoS One. 2012 Apr 2;7(4):e32472. doi: 10.1371/journal.pone.0032472 (PMC3317642; doi:10.1371/journal.pone.0032472)
Supplement: Figure S6 — Rod photoreceptor cell death in ADCY RHO tail (+) is light dependent. (A and B) Animals were reared in constant darkness (A) or in constant light (B). Light exposure reduces the survival of rod photoreceptor cells. Rhodopsin is visualized by antibody (green) and F-actin by phalloidin (red). (Bar = 100 µm.) (C) The number of survived rod photoreceptors in ADCY RHO tail (+) fish under constant darkness (black dots) and under constant light (red dots). (Bars mean SD, * means p<0.05.) (DOC) [file pone.0032472.s006.doc]

Figure S6. Rod photoreceptor cell death in ADCY RHO tail (+) is light dependent.


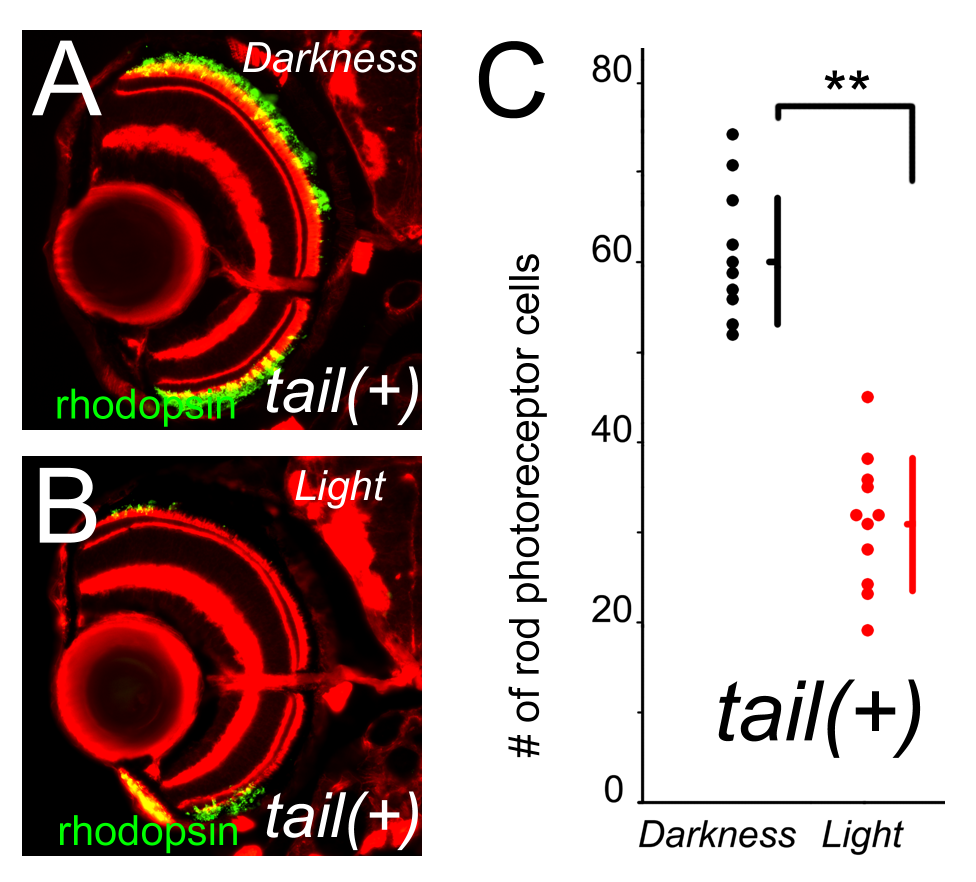


(A and B) Animals were reared in constant darkness (A) or in constant light (B). Light exposure reduces the survival of rod photoreceptor cells. Rhodopsin is visualized by antibody (green) and F-actin by phalloidin (red). (Bar = 100 µm.)

(C) The number of survived rod photoreceptors in ADCY RHO tail (+) fish under constant darkness (black dots) and under constant light (red dots). (Bars mean SD, * means p < 0.05.)
