# Supplementary material for: The Role of Mislocalized Phototransduction in Photoreceptor Cell Death of Retinitis Pigmentosa
Source: PLoS One. 2012 Apr 2;7(4):e32472. doi: 10.1371/journal.pone.0032472 (PMC3317642; doi:10.1371/journal.pone.0032472)
Supplement: Figure S7 — Cones are not decreased in ADCY RHO tail (+) fish. (A and B) Sections of eyes from wt (A) and ADCY RHO tail (+) fish (B) at 7 dpf. R/G cone photoreceptors were visualized with zpr1 (green) and F-actin by phalloidin (red). (Bar = 100 µm) No significant difference was observed under the normal light condition. (C) The number of R/G cone photoreceptors in wt (black dots) and ADCY RHO tail (+) fish (red dots) at 7 dpf. (Bars mean SD.) (DOC) [file pone.0032472.s007.doc]

Figure S7. Cones are not decreased in ADCY RHO tail (+) fish.

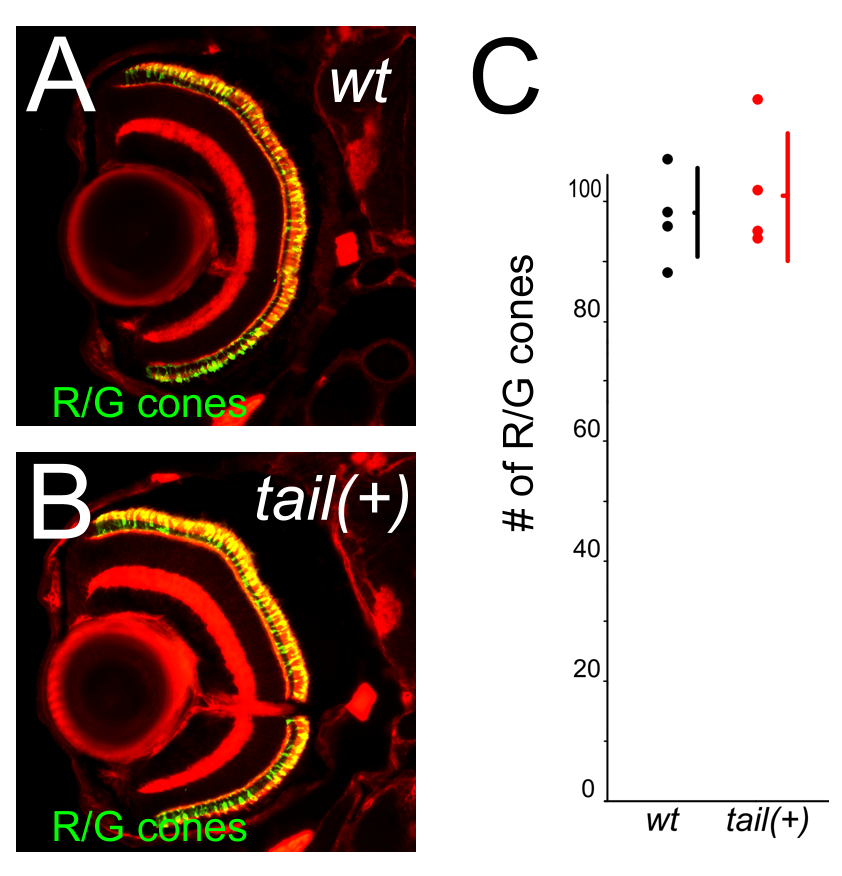


(A and B) Sections of eyes from wt (A) and ADCY RHO tail (+) fish (B) at 7 dpf. R/G cone photoreceptors were visualized with zpr1 (green) and F-actin by phalloidin (red). (Bar = 100 µm) No significant difference was observed under the normal light condition.

(C) The number of R/G cone photoreceptors in wt (black dots) and ADCY RHO tail (+) fish (red dots) at 7 dpf. (Bars mean SD.)
