# Supplementary material for: The Role of Mislocalized Phototransduction in Photoreceptor Cell Death of Retinitis Pigmentosa
Source: PLoS One. 2012 Apr 2;7(4):e32472. doi: 10.1371/journal.pone.0032472 (PMC3317642; doi:10.1371/journal.pone.0032472)
Supplement: Figure S8 — Inner nuclear layer is not affected by 8-Bromo-cAMP treatment. (A and B) Eye sections at 5 dpf ovl treated with 10 µM of a cAMP analogue, 8-Bromo-cAMP (B) and control (A). There are no significant differences. (C) The thickness of inner nuclear layer was not affected by 8-Bromo-cAMP treatment. (DOC) [file pone.0032472.s008.doc]

Figure S8. Inner nuclear layer is not affected by 8-Bromo-cAMP treatment


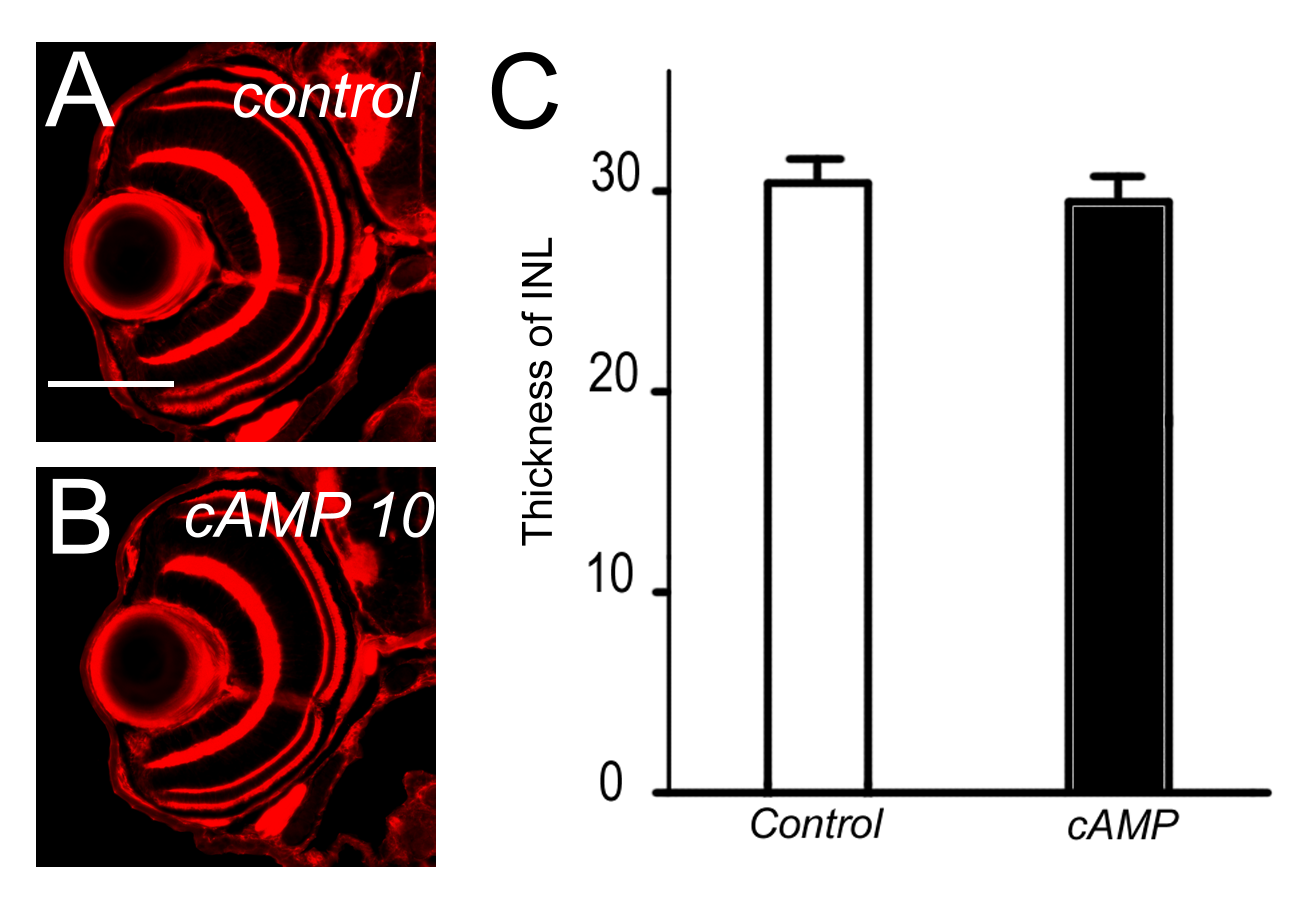


(A and B) Eye sections at 5 dpf *ovl* treated with 10 µM of a cAMP analogue, 8-Bromo-cAMP (B) and control (A). There are no significant differences. (C) The thickness of inner nuclear layer was not affected by 8-Bromo-cAMP treatment.
