# Supplementary material for: The Role of Mislocalized Phototransduction in Photoreceptor Cell Death of Retinitis Pigmentosa
Source: PLoS One. 2012 Apr 2;7(4):e32472. doi: 10.1371/journal.pone.0032472 (PMC3317642; doi:10.1371/journal.pone.0032472)
Supplement: Figure S9 — Western blotting of wild type and the ovl fish with pCREB antibody. Western blot of phosphorylated CREB (A) and β-actin (B) in ovl eye. Anti-CREB antibodies did not work in zebrafish, so we used β-actin as a control. The numbers below the blot is the raw densitometry data. pCREB is increased 6.37% under the normalization by β-actin. (DOC) [file pone.0032472.s009.doc]

Figure S9. Western blotting of wild type and the *ovl* fish with pCREB antibody.


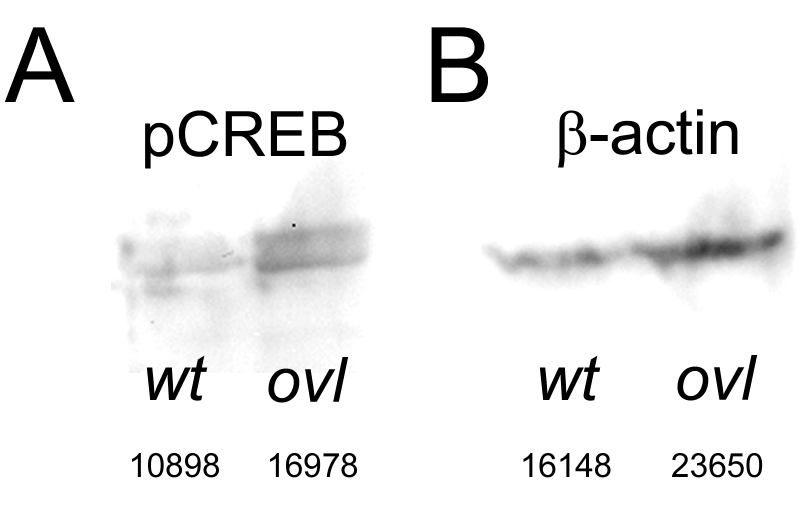


Western blot of phosphorylated CREB (A) and β-actin (B) in ovl eye. Anti-CREB antibodies did not work in zebrafish, so we used β-actin as a control. The numbers below the blot is the raw densitometry data. pCREB is increased 6.37 % under the normalization by β-actin.
