# Supplementary material for: The Role of Mislocalized Phototransduction in Photoreceptor Cell Death of Retinitis Pigmentosa
Source: PLoS One. 2012 Apr 2;7(4):e32472. doi: 10.1371/journal.pone.0032472 (PMC3317642; doi:10.1371/journal.pone.0032472)
Supplement: Figure S11 — Cones do not significantly degenerate in rhodopsin Q344X transgenic fish at 1.5 mpf. (A and B) Sections of eyes from wt (A) and Q344X transgenic fish (B) at 1.5 mpf. UV cone photoreceptors were visualized with EGFP (green) and rhodopsin by antibody (red). OS: outer segment, IS: inner segment. (DOC) [file pone.0032472.s011.doc]

Figure S11. Cones do not significantly degenerate in rhodopsin Q344X transgenic fish at 1.5 mpf.


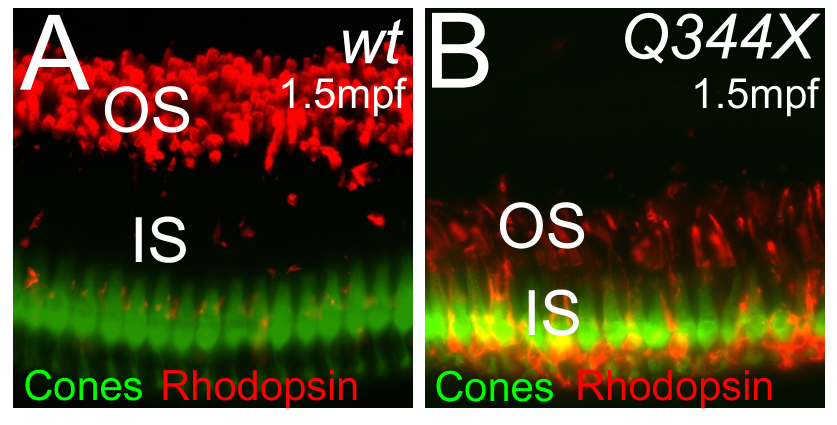


(A and B) Sections of eyes from wt (A) and Q344X transgenic fish (B) at 1.5 mpf. UV cone photoreceptors were visualized with EGFP (green) and rhodopsin by antibody (red). OS: outer segment, IS: inner segment
